# Testing a new platform to screen disease-modifying therapy in type 1 diabetes

**DOI:** 10.1371/journal.pone.0293268

**Published:** 2023-12-14

**Authors:** Sandra M. Lord, Henry T. Bahnson, Carla J. Greenbaum, David R. Liljenquist, John Virostko, Cate Speake

**Affiliations:** 1 Center for Interventional Immunology, Benaroya Research Institute at Virginia Mason, Seattle, WA, United States of America; 2 Rocky Mountain Diabetes Center, Idaho Falls, ID, United States of America; 3 Dell Medical School, University of Texas at Austin, Austin, TX, United States of America; University of Montenegro-Faculty of Medicine, MONTENEGRO

## Abstract

Studies of new therapies to preserve insulin secretion in early type 1 diabetes require several years to recruit eligible subjects and to see a treatment effect; thus, there is interest in alternative study designs to speed this process. Most people with longstanding type 1 diabetes no longer secrete insulin. However, studies from pancreata of those with longstanding T1D show that beta cells staining for insulin can persist for decades after diagnosis, and this is paralleled in work showing proinsulin secretion in individuals with longstanding disease; collectively this suggests that there is a reserve of alive but “sleeping” beta cells. Here, we designed a novel clinical trial platform to test whether a short course of therapy with an agent known to have effects in type 1 diabetes with residual endogenous insulin could transiently induce insulin secretion in those who no longer produce insulin. A therapy that transiently “wakes up” sleeping beta cells might be tested next in a fully powered trial in those with endogenous insulin secretion. In this three-arm non-randomized pilot study, we tested three therapies known to impact disease: two beta-cell supportive agents, liraglutide and verapamil, and an immunomodulatory agent, golimumab. The golimumab treated arm was not fully enrolled due to uncertainties about immunotherapy during the COVID-19 pandemic. Participants had mixed-meal tolerance test (MMTT)-stimulated C-peptide below the quantitation limit (<0.02 ng/mL) at enrollment and received 8 to 12 weeks of therapy. At the completion of therapy, none of the individuals achieved the primary outcome of MMTT-stimulated C-peptide ≥ 0.02 ng/mL. An exploratory outcome of the verapamil arm was MRI-assessed pancreas size, diffusion, and longitudinal relaxation time, which showed repeatability of these measures but no treatment effect. The liraglutide and golimumab arms were registered on clinicaltrials.gov under accession number NCT03632759 and the verapamil arm under accession number NCT05847413.

**Trail registration:** Protocols are registered in ClinicalTrials.gov under accession numbers NCT03632759 and NCT05847413.

## Introduction

**Fig 1:**
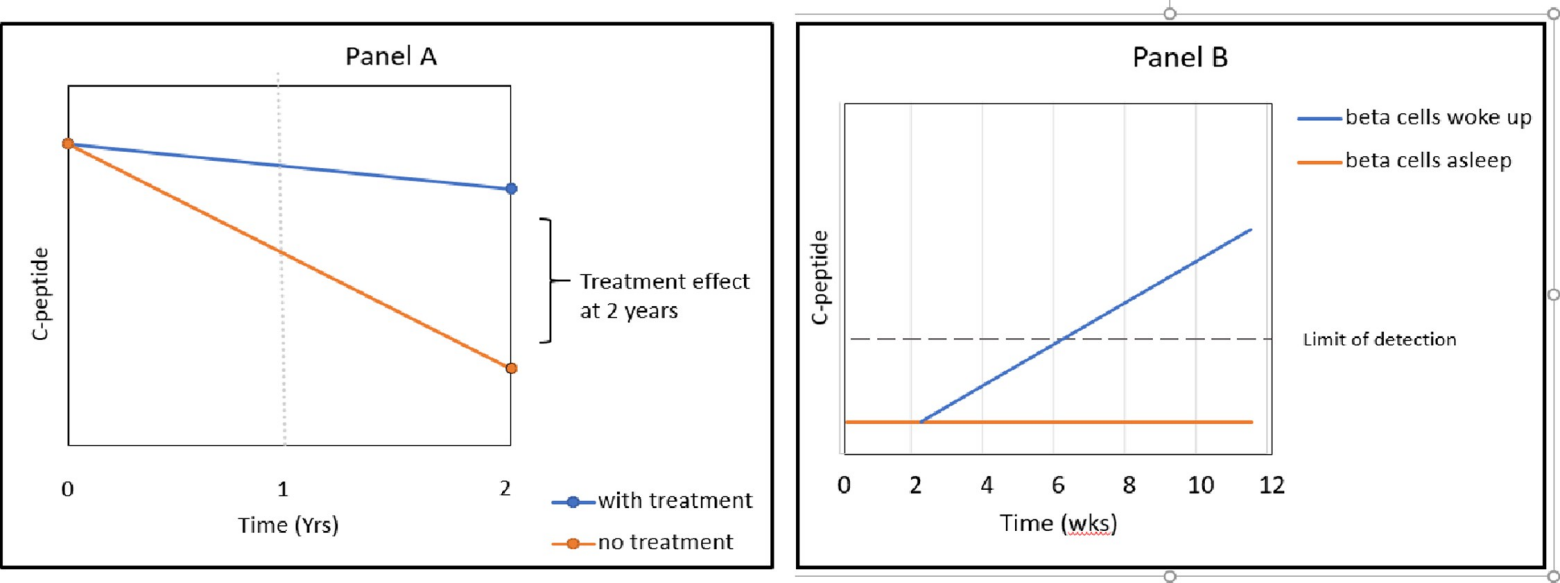
Panel A shows the current paradigm for testing disease modifying therapy in subjects capable of insulin secretion. Panel B shows a novel platform for testing therapies in subjects no longer capable of insulin secretion.

The current paradigm for testing new disease modifying therapy in subjects capable of insulin secretion (C-peptide positive) is illustrated in Fig 1 Panel A. This paradigm requires large subject numbers and typically many years of enrollment to demonstrate a treatment effect. These studies typically use a standard metabolic outcome measure, change in C-peptide level in drug-treated vs placebo-treated groups. The relatively low incidence of T1D [1] lengthens enrollment times, and the high frequency of pediatric diagnosis also impacts trial enrollment since most trials have a regulatory requirement to enroll a population of adults for safety assessments prior to enrollment of children. The time and cost associated with fully-powered randomized controlled trials (RCT) in new onset T1D means that relatively few such trials are ongoing at any given time, slowing the pace of research and the number of therapies that can be tested.

The ability to pre-screen therapies using smaller, single-arm trials in the much larger prevalent population of those with longstanding disease is theoretically appealing. Here, we tested whether therapies could or should be pre-screened in a novel way, where a positive result in a small trial could indicate merit for testing in a larger, fully-powered RCT. Fig 1 Panel B depicts a novel platform for testing new therapies in subjects no longer capable of insulin secretion. Although this population is C-peptide negative, several studies have identified insulin- and proinsulin-positive beta cells in pancreata of those with longstanding T1D [2]. Proinsulin also has been detected in a reasonable proportion of subjects living with T1D for many years [3]. Finally, a study of individuals with T1D receiving a short course of the calcineurin inhibitor rapamycin as pre-conditioning therapy prior to islet transplant showed an increase in insulin secretion in those with undetectable C-peptide [4]. All of this suggests the potential for living but “sleeping” beta cells. A transient change in C-peptide from undetectable (sleeping) to detectable (awake) following a brief intervention could be sufficient to generate interest in a fully powered trial in a population capable of insulin secretion.

Additionally, it would suggest that even those who are C-peptide negative might benefit from disease modifying therapy.

For this pilot study, we examined three different therapies known to either stimulate or preserve insulin secretion in people with diabetes and residual C-peptide: namely, liraglutide, a GLP-1R agonist; verapamil, a calcium channel blocker; and golimumab, a TNF-α blocking agent. These three agents were selected due to their differing mechanisms of action, previously demonstrated safety profiles, and clinical data supporting testing in longstanding type 1 diabetes.

Liraglutide, a GLP-1R agonist, is FDA-approved for treatment of type 2 diabetes, obesity and for secondary prevention of myocardial infarction, stroke and cardiovascular death in people with type 2 diabetes and established cardiovascular disease. As a therapeutic class, the GLP-1/GLP-1R agonists have multiple benefits in type 2 diabetes, including improved beta cell function as evaluated by homeostasis model assessment (HOMA)-B analysis [5, 6], improved proinsulin: insulin ratio [6, 7], enhanced first- and second-phase insulin secretion, and improved beta cell sensitivity to glucose [5, 6]. While GLP-1 agents improve glycemic control in new-onset and established type 1 diabetes [8, 9], information on their effects on beta cell function in those with severely dysfunctional beta cells or undetectable C-peptide are scant and inconsistent [9–11]. For our study, an eight-week treatment duration was selected based on the phase 3 ADJUNCT-one trial [8], which randomized 1398 adults with type 1 diabetes duration ≥ 12 months and variable glycemic control to one of four treatment groups: placebo, liraglutide 0.6 mg daily, liraglutide 1.2 mg daily, or liraglutide 1.8 mg daily for 52 weeks. While the primary outcome was the effect of liraglutide added to insulin therapy on glycemic control and other clinical measures as measured after 52 weeks of treatment, there was a treatment effect (significantly lower HbA1c) in the 1.2 mg and 1.8 mg treated groups compared to placebo and 0.6 mg group at just eight weeks of therapy.

Verapamil is an FDA-approved calcium channel blocker that decreases the expression of thioredoxin-interacting protein (TXNIP), a protein suggested to have deleterious effects on beta cell function [12, 13]. Pre-clinical and clinical data suggest that verapamil has a positive impact on beta cell function in those capable of C-peptide secretion [13], including a recent fully powered RCT showing that verapamil could preserve insulin secretion in newly-diagnosed children [14]. An earlier pilot phase 2 study conducted in people with recent onset type 1 diabetes demonstrated that subjects treated with oral verapamil had slower C-peptide decline over 12 months [15]. At 12 weeks, the stimulated C-peptide AUC was significantly higher in the verapamil-treated group compared to placebo-treated subjects (P = 0.0334), which informed the 12-week treatment duration for our study.

Golimumab is a fully human monoclonal antibody that binds TNF-α with high affinity and specificity and is approved to treat ulcerative colitis and rheumatologic disorders, including rheumatoid arthritis. TNF-α may promote diabetes autoimmunity by enhancing the recruitment of inflammatory cells to the islets, activating cells, and enhancing autoantigen presentation [16, 17]. TNF-α blocking agents have demonstrated efficacy in preserving insulin secretion in new onset type 1 diabetes; initially in a pilot study of etanercept [18] and more recently, in a phase 1/2 clinical trial of golimumab [19, 20]. For our study, the eight-week treatment duration was informed in part by the etanercept pilot study that showed a significant treatment effect (relative change in HbA1c) after eight weeks of therapy.

Here, we tested liraglutide, verapamil, and golimumab in small studies of individuals with longstanding T1D to determine whether any of these would transiently induce insulin secretion.

An exploratory outcome of the verapamil arm was MRI-assessed pancreas size, diffusion, and relaxivity in a population with long standing type 1 diabetes. Pancreatic volume as measured by MRI may be an alternative measure of type 1 diabetes disease state and progression. Recent studies demonstrate reduced pancreatic volume in patients newly diagnosed with type 1 diabetes and in autoantibody positive individuals prior to diagnosis [21–24], although with significant intersubject variability. These findings are interesting given that the beta cells constitute just 1–2% of total pancreatic volume. Changes in pancreatic volume or altered MRI- measured diffusion [24] might indicate disease state or predict response to therapy. In the verapamil arm of our study, eight of 10 subjects underwent optional pancreatic MRIs before and after 12 weeks of verapamil treatment to assess pancreas size, diffusion, and relaxivity, in addition to intersubject and intrasubject variability of these measures.

## Research design and methods

### Trial enrollment

**Fig 2:**
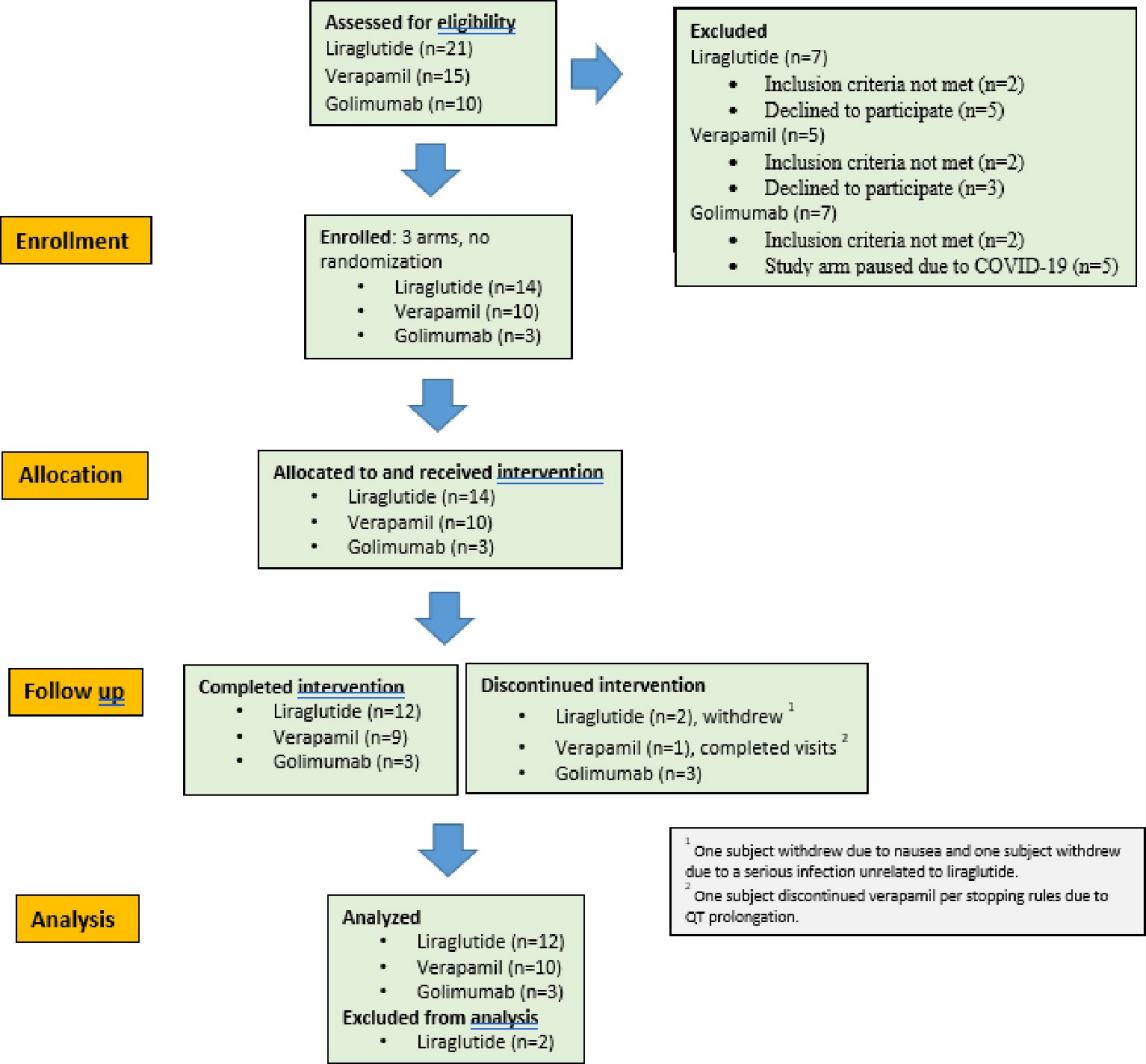
CONSORT diagram representing participant flow in the trial.

Fig 2 is a CONSORT diagram representing participant flow in the trial. This was a three-arm, non-randomized pilot study. The TREND checklist is provided as S1 Checklist. The liraglutide and golimumab arms were registered on clinicaltrials.gov under accession number NCT03632759; please see S1 Protocol for the clinical study protocol. There were two clinical sites for this protocol: Benaroya Research Institute at Virginia Mason Medical Center in Seattle, WA and Rocky Mountain Diabetes Center in Idaho Falls, ID. When the golimumab arm was paused during COVID-19, we submitted a second protocol to test verapamil. Please see S2 Protocol for the verapamil clinical study protocol. The verapamil arm was registered on clinicaltrials.gov under accession number NCT05847413. The verapamil study was not submitted to clinicaltrials.gov until after enrollment was completed, which was a study team oversight. The authors confirm that all ongoing and related trials are registered. Benaroya Research Institute was the only clinical site for the verapamil protocol. Both study protocols were approved by the Benaroya Research Institute IRB under protocol numbers IRB18004 and IRB20042. Both protocols were conducted according to the principles expressed in the Declaration of Helsinki. Subjects were self-referred or referred from local endocrinology offices. An eligibility phone call was conducted by a study provider prior to the screening visit. All subjects provided written informed consent at the screening visit. All visits were conducted in person at the BRI Clinical Research Center or at the Rocky Mountain Diabetes Center by appropriately trained research staff (providers, registered nurses and study coordinators). Recruitment activities and all study visits were conducted between August 2018 and December 2021. Some authors (those who were involved in clinical oversight of study subjects) had access to information that could identify individual participants during and after data collection. We included subjects aged 18–50, type 1 diabetes duration > 10 years, hemoglobin A1c ≤ 8.5%, otherwise healthy, and with undetectable peak C-peptide (<0.02 ng/mL) on two consecutive 2-hour MMTTs. There were exclusions related to specific risks of each arm; for example, subjects with a history of pancreatitis were excluded from the liraglutide arm, subjects with QT prolongation were excluded from the verapamil arm, subjects with claustrophobia were excluded from the optional MRI of the verapamil arm, and subjects with significant leukopenia were excluded from the golimumab arm. Use of non-insulin therapies to control hyperglycemia or use of medications known to influence glucose tolerance such as corticosteroids were exclusionary for all arms. Subjects were permitted to participate in more than one study arm if eligible, with a minimum washout period between arms of three months. Mean age and duration of disease varied slightly depending on the study arm. See Table 1 for a description of the baseline characteristics of each cohort.

**Table 1:** Baseline characteristics of subjects.

Study Arm	male/female	Mean (range) BMI at baseline in kg/m^2^	Mean (range) age in years	Mean (range) duration of T1D in years
Liraglutide N = 14	4/10	30.19	41 (24–49)	28.8 (10.4–43.1)
Verapamil N = 10	3/7	27.04	29 (22–50)	22.6 (2.0–46.9)
Golimumab N = 3	0/3	30.07	45.3 (44–46)	34.2 (33.3–34.0)

Liraglutide was tested first and given as an escalating subcutaneous daily dose from 0.6 mg for one week, then 1.2 mg for one week, then 1.8 mg for six weeks. Most doses were administered at home by the study subject, after appropriate training by study staff. Verapamil was tested next and administered as an escalating oral daily dose starting with 120 mg for two weeks, then 240 mg for two weeks, then 360 mg for the remainder of the twelve-week treatment period. Most doses were administered at home by the study subject. Golimumab was tested last and administered subcutaneously every two weeks: 100 mg for the first two doses, then 50 mg for eight weeks total. Most doses were administered at home by the study subject, after appropriate training by study staff. The golimumab arm was not completed due to uncertainties about immunotherapy during the COVID-19 pandemic. For all three arms, subjects were asked to keep a medication log to document administration time, location of injection if relevant, and any side effects.

The primary outcome measure was binary; a positive result was transition from MMTT-stimulated C-peptide negative (<0.02 ng/mL) to C-peptide positive ≥ 0.02 ng/mL at treatment end in 20% of participants. Up to 45 people (15 in each drug arm) were targeted for enrollment. This target enrollment was based on statistical modeling suggesting 15 subjects would give 83% power to detect a positive primary outcome in 20% of participants, a cutoff that seemed achievable, clinically relevant, and sufficient to warrant testing the drug in a larger population.

The verapamil arm had an exploratory outcome measure: MRI measured pancreatic size in subjects with longstanding T1D and the change in pancreatic volume and relationship of change in pancreas size, diffusion, and relaxivity to response to 12 weeks of verapamil therapy.

### Laboratory measurements

C-peptide was measured using a Tosoh assay at the University of Florida Health Pathology Laboratory Endocrine Autoantibodies/Diabetes Central Laboratory. All other laboratory values were run at the study site; either Virginia Mason Medical Center, Seattle, WA or Rocky Mountain Diabetes Center in Idaho Falls, ID.

### MRI measurements

All MRIs were conducted on BRI-consented subjects using 1.5 Tesla scanners located at Virginia Mason Medical Center. The MRI protocol consisted of a quantitative abdominal protocol previously validated for multisite reproducibility [25]. De-identified MRI data was shared with the University of Texas Austin for pancreas MRI analysis. MRI data was coded such that the image reader was blinded to any information on treatment status or any link between repeat scans in an individual. For each MRI scan, the pancreas was outlined freehand on contiguous slices of the anatomical image. This resulting pancreas mask was multiplied by the image resolution to calculate pancreas volume and then divided by the subject weight to yield the pancreas volume index. The pancreas mask was multiplied by parametric maps of the apparent diffusion coefficient and longitudinal relaxation time (T1) to extract mean pancreatic values for each parameter.

### Statistical analysis

Conditional power analysis was performed as described [26–28]. In the conditional power analysis, we aimed to detect a difference of 20% between the null proportion of 0% and the alternative proportion of 20% at a significance level of 0.05 using a one-sided one-sample z-test. Other inputs to the conditional power analysis included the anticipated total sample size (N = 15), current sample size of 10 at the time of the conditional power analysis, P0 the proportion assuming the null hypothesis (0%), P1 the proportion assuming the alternative hypothesis (20%), alpha the probability of rejecting a true null hypothesis (0.05), and futility equaling one minus the conditional power. For the MRI reliability analysis, ICC was calculated in Excel using a two-way random effects model. All other analysis was performed using SAS software version 9.4 (SAS Institute Inc., Cary, NC, USA) and JMP Pro 16 (SAS Institute Inc., Cary, NC, USA).

## Results

### Metabolic results

No individual in any of the therapeutic arms showed detectable insulin secretion at the end of the treatment window (Table 2); none of the trials met their primary endpoint. Individual-level metabolic data are provided in S1 Data. While we originally aimed to enroll 15 people in each arm of the study, a conditional power analysis was conducted after the first 10 subjects completed the liraglutide arm and no individuals had C-peptide ≥ 0.02 ng/mL. This analysis indicated there was only 9% power to meet the primary outcome, or a 91% futility index for the liraglutide arm. Completion of the last two study subjects (already on liraglutide at the time of the conditional power analysis) reduced this power even further. The same conditional power/futility index was applied to the verapamil arm; for this reason, these studies were stopped after 12 and 10 subjects, respectively. The golimumab arm of the study was stopped after enrollment of only three individuals due to uncertainties around safety of immunotherapy early in the COVID-19 pandemic.

**Table 2:** Metabolic results for each therapy.

Study arm	Mean peak stimulated C-peptide at baseline	Mean peak stimulated C-peptide at endpoint^a^	Mean HbA1c at baseline^b^	Mean HbA1c at endpoint^b^^,^^c^
Liraglutide	< 0.02 ng/mL N = 14	< 0.02 ng/mL N = 12	6.8 (51); 5.1–7.5 (32–58) N = 14	6.7 (49.7); 5.7–7.4 (39–57) N = 7
Verapamil	< 0.02 ng/mL N = 10	< 0.02 ng/mL N = 10	6.5 (48); 5.6–7.2 (38–55) N = 10	6.6 (48.6); 5.8–7.4 (40–57) N = 8
Golimumab	< 0.02 ng/mL N = 3	< 0.02 ng/mL N = 3	6.6 (48.6); 6.1–7.0 (43–53) N = 3	6.5 (48); 6.3–6.9 (45–52)N = 3

^a^Includes subjects who completed the study.

^b^Summaries presented as mean % (mean mmol/mol); min/max range in % (min/max range in mmol/mol).

^c^Five of 12 liraglutide HbA1cs were not collected at the final study visit. Two of 10 verapamil HbA1cs were not collected at the final study visit.

The Fig 2 CONSORT diagram illustrates participant flow in the trial. Fourteen subjects were allocated to receive liraglutide. One subject withdrew due to nausea and decreased appetite, and a second due to a serious genital infection unrelated to liraglutide. Twelve subjects completed liraglutide treatment. None of the remaining 12 subjects had a peak C-peptide level ≥ 0.02 ng/mL eight weeks after initiation of liraglutide treatment. There was no significant change in mean HbA1c at the final study visit. Non-serious adverse events included nausea (13/14 subjects), decreased appetite (12/14), weight loss (9/14), gastroesophageal reflux/heartburn (5/14) and injection site reactions (5/14).

Ten subjects were allocated to receive verapamil. One subject had an abnormal EKG (prolonged QT interval) at the week six visit and verapamil was discontinued per pre-specified dose withholding rules; the subject completed all remaining study visits. None of the subjects had a peak C-peptide level ≥ 0.02 ng/mL after 12 weeks of verapamil treatment. There was no significant change in mean HbA1c at the final study visit. Non-serious adverse events included constipation, which was reported by 5/10 subjects.

Three subjects were allocated to receive golimumab and three subjects completed treatment. None of the three subjects had a peak C-peptide level ≥ 0.02 ng/mL eight weeks post-golimumab initiation. There was no significant change in mean HbA1c at the final study visit. Non-serious adverse events included upper respiratory infection, acute cystitis, and injection site bruising.

### MRI results

**Fig 3:**
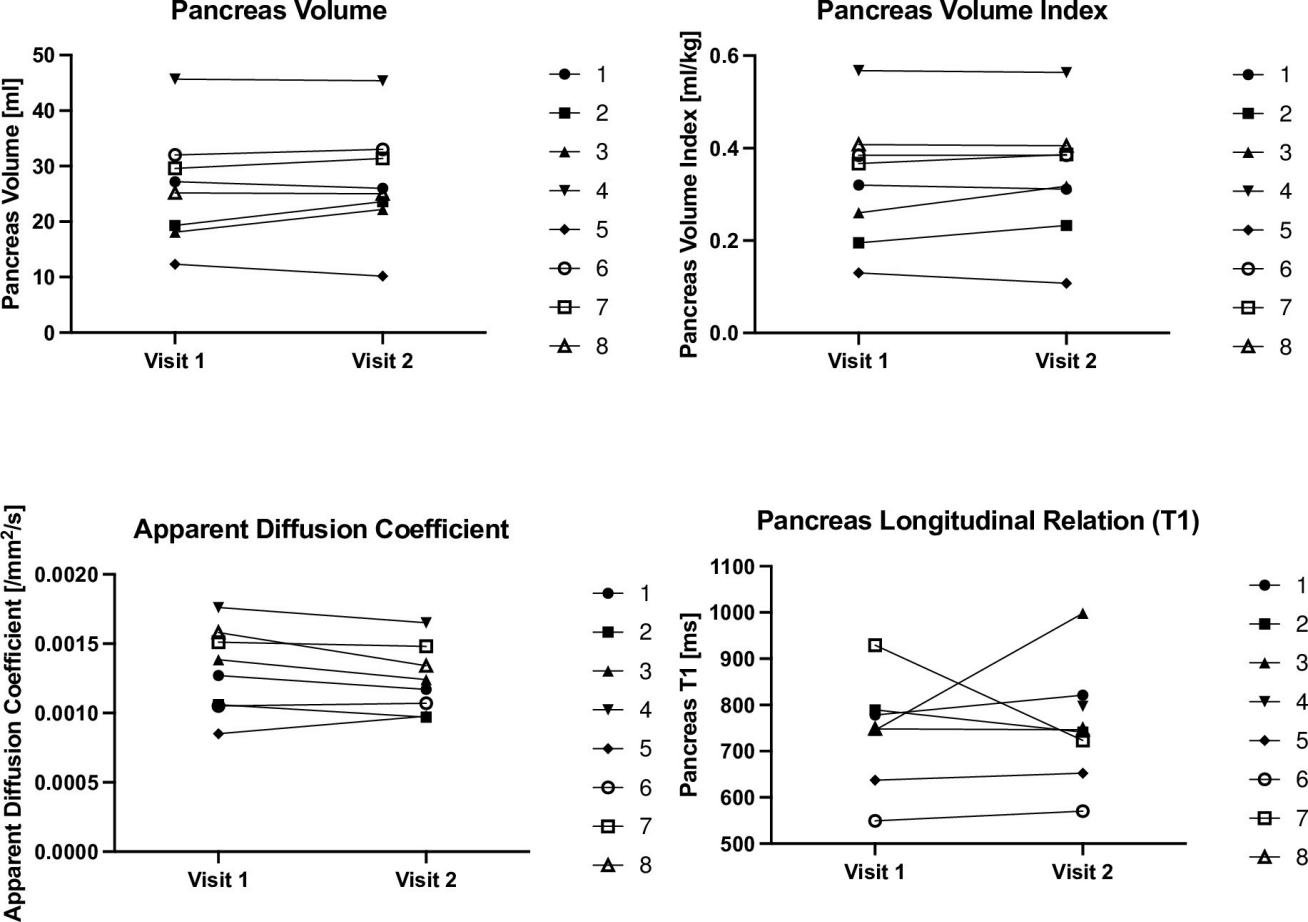
Shows pancreas size, diffusion and longitudinal relaxation time.

In the eight subjects studied, all from the verapamil arm, MRI measures of pancreas size, diffusion, and longitudinal relaxation time showed no clear treatment response, as shown in Fig 3 and detailed in S2 Data.

Pancreas volume, pancreas volume index, and apparent diffusion coefficient all showed excellent reliability, with intraclass correlation coefficients (ICC) of 0.97, 0.98, and 0.90, respectively. Pancreas longitudinal relaxation time (T1) exhibited a poor reliability (ICC = 0.47), likely due to respiratory motion affecting this image acquisition.

## Discussion

This project proposed a novel template for rapid testing of disease modifying therapy in subjects with longstanding type 1 diabetes who are making little or no C-peptide. This study utilized a novel binary outcome measure, a transition from C-peptide negative to positive in those potentially still capable of insulin secretion. This contrasts with the standard outcome measure of a disease-modifying intervention in type 1 diabetes; that is, change in C-peptide level with and without intervention, a measure that generally requires a prolonged recruitment period and sustained follow-up. Our study population and outcome measure were designed to efficiently screen therapies using a short treatment period and small sample size. Although liraglutide, verapamil, and golimumab have each demonstrated benefits in subjects with type 1 diabetes and residual insulin secretion, we found no evidence that short-term treatment with these agents stimulates transient insulin secretion in people with C-peptide negative type 1 diabetes. Of note, one of 10 participants studied in the verapamil arm experienced QT prolongation, requiring withholding of study drug.

While sufficient individuals were studied in the liraglutide and verapamil cohorts to address the question, an important caveat to interpretation of the golimumab result is that only three individuals were studied. Further, it is possible that more prolonged treatment or different dosing would have been effective for any of the therapies. More likely, however, our results suggest that the mechanisms that result in beta cell dysfunction/destruction early after diagnosis are different in type and/or degree as compared to those with longstanding disease. MRI assessment of pancreas size, diffusion, and relaxivity in this population with long-standing T1D before and after treatment found no treatment effect, but MRI measures of pancreas size and diffusion were repeatable, with minimal intrasubject variability.

## Conclusion

Our results suggest that this platform is unlikely to be useful to select therapies to be tested further in a fully powered trial in recent onset type 1 diabetes. In this pilot study of subjects with long-standing T1D, MRI assessed measures of pancreas size and diffusion appear repeatable.

## Supporting information

S1 Checklist TREND checklist. (PDF)

S1 Protocol Liraglutide/golimumab protocol. (DOCX)

S2 Protocol Verapamil protocol. (DOCX)

S1 Data Metabolic data. (XLSX)

S2 Data MRI data. (XLSX)
