# Supplementary material for: Testing a new platform to screen disease-modifying therapy in type 1 diabetes
Source: PLoS One. 2023 Dec 14;18(12):e0293268. doi: 10.1371/journal.pone.0293268 (PMC10721089; doi:10.1371/journal.pone.0293268)
Supplement: S1 Protocol — (DOCX) [file pone.0293268.s002.docx]

**Protocol**

**Targeting Beta cell Dysfunction in Longstanding T1D**

Short Title: Waking Beta Cells

Version 4.0

19 NOV 2019

Investigator

Carla Greenbaum, MD

Table of Contents

**Section Page**

[1 Abbreviations 7](#_Toc25659931)

[2 Protocol Synopsis 10](#_Toc25659932)

[3 Background and rationale 11](#_Toc25659933)

[4 Study Objectives 16](#_Toc25659934)

[4.1 Primary Objective 16](#_Toc25659935)

[4.2 Secondary Objectives 16](#_Toc25659936)

[4.3 Safety Objective 16](#_Toc25659937)

[5 Study Design 16](#_Toc25659938)

[5.1 Overview 16](#_Toc25659939)

[5.2 Endpoints 17](#_Toc25659940)

[5.2.1 Primary Endpoint 17](#_Toc25659941)

[5.2.2 Secondary/Exploratory Endpoints 17](#_Toc25659942)

[5.3 Eligibility 17](#_Toc25659943)

[5.3.1 Inclusion criteria 17](#_Toc25659944)

[5.3.2 Exclusion criteria 18](#_Toc25659945)

[5.4 Study Duration 19](#_Toc25659946)

[5.5 Study sites 19](#_Toc25659947)

[6 Study Procedures 19](#_Toc25659948)

[6.1 Screening Visits (visit numbers -2,-1) 19](#_Toc25659949)

[6.2 Administration of Study Drug 19](#_Toc25659950)

[6.2.1 Formulation 19](#_Toc25659951)

[6.2.1.1 Liraglutide: (Victoza®; NovoNordisk). 19](#_Toc25659952)

[6.2.1.2 Golimumab: (SIMPONI®; Janssen). 20](#_Toc25659953)

[6.2.2 Preparation and Accountability 20](#_Toc25659954)

[6.2.3 Initial Treatment Visit (visit number 0) 20](#_Toc25659955)

[6.2.4 Dosing Windows 21](#_Toc25659956)

[6.3 Subsequent study visits (visits 1, 2) 21](#_Toc25659957)

[6.4 Follow-up study visit (visit 3) 21](#_Toc25659958)

[6.5 Visit windows 21](#_Toc25659959)

[6.6 Assessments 22](#_Toc25659960)

[6.6.1 General Assessments 22](#_Toc25659961)

[6.6.2 Clinical Laboratory Assessments 22](#_Toc25659962)

[6.6.3 Metabolic Assessments 23](#_Toc25659963)

[6.6.4 Mechanistic Assessments 23](#_Toc25659964)

[6.6.4.1 Mechanistic samples 23](#_Toc25659965)

[7 PARTICIPANT SAFETY 23](#_Toc25659966)

[7.1.1 Benefit of Participation 23](#_Toc25659967)

[7.1.2 Risks of Participation and Mitigation of Risks 23](#_Toc25659968)

[8 Adverse Event Reporting and Documentation 26](#_Toc25659969)

[8.1 Overview 26](#_Toc25659970)

[8.2 Definitions 26](#_Toc25659971)

[8.2.1 Adverse Event (AE) 26](#_Toc25659972)

[8.2.2 Adverse Reaction 26](#_Toc25659973)

[8.2.3 Serious Adverse Event (SAE) 27](#_Toc25659974)

[8.2.4 Unexpected or Unlisted Adverse Event 28](#_Toc25659975)

[8.2.5 Definitions Specific for Study B: Golimumab (SIMPONI®) 28](#_Toc25659976)

[8.2.5.1 Adverse Events of Special Interest for Golimumab (SIMPONI®) 28](#_Toc25659977)

[8.2.5.2 Individual Case Safety Report (ICSR) for Golimumab (SIMPONI®) 28](#_Toc25659978)

[8.2.5.3 Product Quality Complaint (PQC) for Golimumab (SIMPONI®) 29](#_Toc25659979)

[8.3 Safety Assessments and Collection of Safety Data 29](#_Toc25659980)

[8.3.1 Procedures for Reporting to Janssen Scientific Affairs for Golimumab (SIMPONI®) 30](#_Toc25659981)

[8.3.2 SAEs and Special Reporting Situations 30](#_Toc25659982)

[8.3.2.1 Additional SAE Reporting requirements for Golimumab (SIMPONI®) 31](#_Toc25659983)

[8.3.2.2 Additional Special Reporting Situations for Golimumab (SIMPONI®) 31](#_Toc25659984)

[8.3.2.3 Adverse Events of Special Interest for Golimumab (SIMPONI®) 32](#_Toc25659985)

[8.3.2.4 Product Quality Complaints (PQC) for Golimumab (SIMPONI®) 32](#_Toc25659986)

[8.3.3 Reporting pregnancy 32](#_Toc25659987)

[8.3.3.1 Reporting Pregnancy for Golimumab (SIMPONI®) 32](#_Toc25659988)

[9 Statistical Analysis Plan 33](#_Toc25659989)

[9.1 Endpoints: 33](#_Toc25659990)

[9.1.1 Primary Endpoint 33](#_Toc25659991)

[9.1.2 Secondary and Exploratory Endpoints 33](#_Toc25659992)

[9.2 Sample size: 33](#_Toc25659993)

[10 ETHICAL CONSIDERATIONS AND COMPLIANCE WITH GOOD CLINICAL PRACTICE 34](#_Toc25659994)

[10.1 Statement of Compliance 34](#_Toc25659995)

[10.2 Informed Consent 34](#_Toc25659996)

[10.3 Withdrawal of Subjects from the Study 34](#_Toc25659997)

[10.4 Privacy and Confidentiality 35](#_Toc25659998)

[11 Data Collection, Monitoring, and Sample Retention 35](#_Toc25659999)

[11.1 Data Collection Instruments 35](#_Toc25660000)

[11.2 Archival of Data 35](#_Toc25660001)

[11.3 Monitoring 35](#_Toc25660002)

[11.4 Sample Retention 36](#_Toc25660003)

[12 Schedule of assessments 37](#_Toc25660004)

[Study A: Schedule of Assessments for Liraglutide 37](#_Toc25660005)

[Study B: Schedule of Assessments for Golimumab: 38](#_Toc25660006)

[13 References 39](#_Toc25660007)

[14 Attachments 43](#_Toc25660008)

[Attachment 1. Common Terminology Criteria for Adverse Events (CTCAE) Version 5 43](#_Toc25660009)

[Attachment 2. Victoza® (liraglutide) Prescribing Information 43](#_Toc25660010)

[Attachment 3. SIMPONI® (golimumab) Prescribing Information 43](#_Toc25660011)

# Abbreviations

**AE** Adverse event

**ALT** Alanine aminotransferase

**AST** Aspartate aminotransferase

**AUC** Area under the curve

**BRI** Benaroya Research Institute at Virginia Mason

**BUN** Blood urea nitrogen

**CBC** Complete blood count

**CFR** Code of Federal Regulations

**CHF** Congestive heart failure

**CMV**  Cytomegalovirus

**CNS** Central nervous system

**CRF** Case report form

**CRP** C-reactive protein

**CTCAE**  Common Terminology Criteria for Adverse Events

**DHHS** Department of Health and Human Services

**DKA** Diabetic ketoacidosis

**DPP-4** Dipeptidyl peptidase 4

**EBV** Epstein-Barr virus

**ESR** Erythrocyte sedimentation rate

**FDA** US Food and Drug Administration

**GAD-65** Glutamate decarboxylase-65

**GCP** Good clinical practice

**GLP-1** Glucagon-like peptide 1

**GLP-1R**  Glucagon-like peptide 1 receptor

**HbA1c**  Glycosylated hemoglobin

**HBV** Hepatitis B virus

**HCV** Hepatitis C virus

**HDL** High-density lipoprotein

**HIV** Human immunodeficiency virus

**IA-2** Insulinoma antigen-2

**IAPP** Islet amyloid polypeptide

**IB** Investigator's brochure

**ICH**  International Conference on Harmonization

**IL** Interleukin

**IRB** Institutional review board

**LDL** Low-density lipoprotein

**LFT** Liver function test

**MHC** Major histocompatibility complex

**MMTT** Mixed-meal tolerance test

**MTC** Medullary thyroid cancer

**NOD** Non-obese diabetic

**PCR** Polymerase chain reaction

**PI** Proinsulin

**PI/C** Proinsulin/C-peptide

**PK** Pharmacokinetic

**PPD** Purified protein derivative

**pro-IAPP** Prohormone IAPP_1-48_

**RA** Rheumatoid arthritis

**SAE** Serious adverse event

**SC** Subcutaneous

**T1D**  Type 1 diabetes

**TB** Tuberculosis

**TNFα** Tumor necrosis factor alpha

**ULN**  Upper limit of normal range

**VMMC**  Virginia Mason Medical Center

**WBC** White blood cell

**WIRB** Western Institutional Review Board

**ZnT8**  Zinc transporter-8

# Protocol Synopsis

| Title | **Targeting Beta cell Dysfunction in Longstanding T1D** |
| --- | --- |
| Short Title | Waking Beta Cells |
| Sponsor Investigator | Carla Greenbaum, MD |
| Sponsor | Benaroya Research Institute at Virginia Mason |
| Conducted at | Benaroya Research Institute at Virginia Mason  Clinical Research Center  1201 Ninth Ave  Seattle,  WA 98101 |
| Study drug support | Janssen Scientific Affairs provided golimumab (SIMPONI®) |
| Accrual Objective | 15 - 30 subjects |
| Study Treatment | Two months of therapy with liraglutide (Victoza®) and/or golimumab (SIMPONI®) |
| Study Design | This application describes two independent open label, proof of concept studies to determine whether therapeutic interventions can transiently improve beta cell function in those who do or do not secrete proinsulin and little/no C-peptide. |
| Primary Objective | To determine whether two therapeutic interventions can transiently improve beta cell function in those individuals who do or do not secrete proinsulin and with C-peptide <0.017 pmol/mL, as assessed by increased C-peptide (primary outcome) or IAPP production, or a reduction in the PI/C or pro-IAPP/IAPP ratios |
| Primary Endpoints | Proportion of individuals with peak MMTT stimulated C-peptide >0.017 pmol/mL at 8 weeks. |
| Secondary Endpoints | - Changes in proinsulin, proinsulin to C-peptide ratio, IAPP and pro-IAPP between screening and 8 week MMTT - The proportion of those with C-peptide >0.017 pmol/mL at 8 weeks who are above and below that threshold after cessation of therapy - Relationship of metabolic measures to genetic, genomic and immunological response |
| Safety Endpoints | - Adverse events associated with liraglutide or golimumab treatment |
| Major Inclusion Criteria | - M/F 18-50 years of age, inclusive - ≥ 3 years from Diagnosis of T1D |

# Background and rationale

Type 1 diabetes mellitus (T1D) is an immune-mediated disease in which insulin-producing beta cells are completely or almost completely destroyed, resulting in life-long dependence on exogenous insulin. It is a chronic and potentially disabling disease that represents a major public health and clinical concern. The number of patients being diagnosed with T1D is increasing each year and is approaching an epidemic level in many countries (1).

For individuals living with T1D, continuous exogenous insulin therapy is needed to prevent ketoacidosis and other catabolic effects of insulin deficiency, to promote anabolism, and to maintain life. While there have been significant improvements in insulin analogs and insulin delivery systems, such as continuous subcutaneous insulin infusions with wearable pumps, continuous glucose monitoring, and hybrid closed loop systems, normal glucose control, particularly in children, is rarely achieved (2). Moreover, while the frequency of long-term complications is decreasing, individuals with T1D continue to have reduced life expectancy (3–5).

**Disease Modifying Therapy in T1D**

Since the first use of insulin in 1922, treatment of T1D has been targeted at controlling the symptoms and consequences of hyperglycemia. This is analogous to symptom control for individuals with rheumatoid and juvenile arthritis, where the aim of therapy was previously to control pain and/or provide devices to adapt to the presence of disability. Yet, control of symptoms of arthritis - and almost all other autoimmune disease - is no longer the primary aim of treatment; instead, the standard of care is disease-modifying therapy with the intent to fundamentally alter the course of the condition. This approach has dramatically changed the lives of those with these autoimmune diseases. The aim of disease-modifying therapy in T1D is the same: to move away from symptom management (e.g., glycemic control) as the mainstay of treatment and find approaches to alter the disease course – specifically to preserve beta cell function measured by C-peptide. Preservation of C-peptide post clinical diagnosis is associated with less short-term (hypoglycemia) and long-term (retinopathy, renal disease) complications. Islet transplant studies indicate that small amounts of endogenous secretion are insufficient for insulin independence, but do prevent hypoglycemia.

Well-controlled clinical trials of disease modifying therapy in T1D have been largely limited to primary prevention (before development of autoantibodies), treatment at early, asymptomatic stages of disease (when antibodies are present with normal or abnormal glucose tolerance), or shortly after the onset of symptoms. At this time, four therapies with reasonable safety profiles have phase 2 clinical trial results demonstrating an altered disease course measured by a reduced fall in C-peptide during a mixed meal tolerance test (MMTT) in individuals shortly after onset of clinical disease (6–9). Each of these are immune therapies. There are no phase 2 controlled trial results currently available using therapies aimed at the beta cell itself to alter disease course at any stage of disease; yet there is increasing interest in testing such agents.


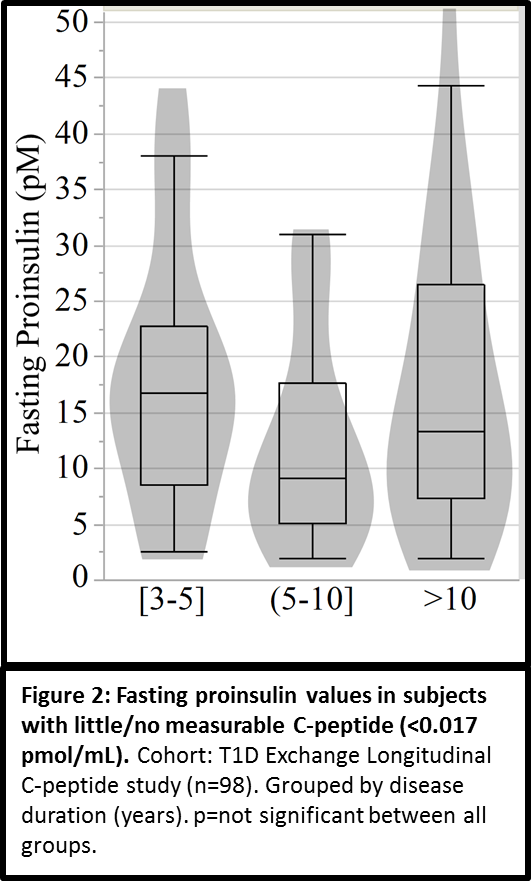
An alternative is to consider enrolling individuals further from diagnosis as there is a large population of adult individuals with longstanding T1D. This group has not previously been enrolled in trials of disease- modifying therapy because, until recently, there has been insufficient longitudinal information about the natural history of insulin secretion years from diagnosis to plan a trial; there has also been a lack of suitable alternative outcomes. We have just completed a longitudinal study of hundreds of individuals with longstanding type 1 diabetes examining the rate of fall of C-peptide assessed by regular MMTT over a 4 year period (manuscript in preparation). Not unexpectedly, among those with C-peptide at the start of the study, the rate of fall in this population is very low; thus, a very large number of participants would have to be studied over many years to detect an effect of therapy on preservation of C-peptide. Thus, one cannot conduct a small or short-term study of disease modifying therapy in those with longstanding T1D using preservation of C-peptide as an outcome.

Excitingly, however, our observation that almost all of the individuals three or more years from diagnosis with below detection limit C-peptide on MMTT secrete significant amounts of proinsulin suggests an alternative outcome measure that could allow for assessing the impact of therapies on beta cell health even in small studies. Since there are hundreds of thousands of individuals with longstanding T1D, if successful, this approach may allow for more rapid advancement of disease-modifying therapies.

We found that of 98 subjects with longstanding T1D and MMTT C-peptide <0.017 pmol/mL, 83 (85%) were secreting proinsulin above the limit of quantitation (2 pM). Proinsulin secretion was not associated with disease duration in this population (*Figure 2*). These data, which parallel recent findings in pancreata from T1D subjects (10), indicate the presence of beta cells (either residual, de-differentiated, or proliferating) in longstanding disease, but that they are not functioning properly. The data raise the question as to whether therapy can improve beta cell dysfunction in this population of subjects who have what we characterize as “sleeping” beta cells. Importantly, testing therapies in this population of individuals who have beta cells that are clearly present but severely dysfunctional or de-differentiated allows for a more direct assessment as to whether such cells are capable of secreting C-peptide. Evidence that this approach is of interest is provided in a study of longstanding T1D subjects awaiting islet transplant (median disease duration 27 years), many of whom showed increased C-peptide levels upon rapamycin induction therapy (11).

To better understand the health of the beta cell, we and others have been measuring proinsulin with the rationale that an increased proinsulin/insulin (or C-peptide) ratio implies defective prohormone processing and could be reflective of beta cell stress (12). The insulin processing pathway involves prohormone convertases 2 and 1/3 (13,14). Impaired processing of proinsulin has been reported in type 2 diabetes (15), islet transplantation (16), and new-onset T1D (17). Studies from Belgium (18), Finland (19) and TrialNet (20) report impaired processing in antibody positive individuals prior to clinical T1D. Importantly, the proinsulin/C-peptide ratio has also been successfully used as an outcome measure in clinical interventions aimed to improve beta cell health. With respect to immunotherapy, in new-onset placebo-treated T1D patients, the fasting proinsulin/C-peptide ratio increased over time while this did not occur in those treated with cyclosporine (21), and anakinra (IL-1RA) treatment in type 2 patients significantly decreased the proinsulin/insulin ratio (22). A decrease in proinsulin/C-peptide ratio was also seen after gastric bypass surgery in obese adolescents coincident with an improved disposition index (beta cell function in relation to insulin sensitivity) (23). These studies support the notion that measurement of proinsulin/C-peptide in response to therapy will be indicative of improved beta cell function.

Other prohormones can also reflect beta cell stress and presence; like insulin/proinsulin, a pro-form of amylin (also known as islet amyloid polypeptide or IAPP) is also detectable in blood and associated with dysfunctional protein processing in the beta cell. Processing of prohormone IAPP_1-48_ (pro-IAPP) to IAPP involves the same enzymes as proinsulin processing - prohormone convertases 2 and 1/3 (24,25). In longstanding T1D subjects from our adult cohort and a Canadian pediatric cohort, the pro-IAPP/IAPP ratio is increased compared to healthy subjects (26). Importantly, pro-IAPP was detectable in 100% of T1D subjects tested. In addition, like proinsulin, it is detectable in subjects without detectable fully processed hormone: 14/33 longstanding T1D subjects tested had detectable pro-IAPP in the absence of detectable IAPP (26). This suggests that inducing/increasing IAPP, or reducing the pro-IAPP/IAPP ratio, like proinsulin/C-peptide, may also reflect improvement of beta cell function in subjects with longstanding disease.

**Interventions:**

There are many therapies that have been proposed to improve beta cell function, by acting directly on the beta cell or by helping to improve the immune or metabolic milieu in the pancreas. Having identified a population of individuals with longstanding T1D who have severely dysfunctional or possibly de-differentiated, but not dead beta cells, one potentially can use this population in pilot studies to evaluate the impact of a series of therapies on beta cell function. In this protocol, we propose to test one therapy primarily targeting the immune system (anti-TNFα) and one primarily acting directly upon the beta cell (GLP-1 agonist). These agents were selected due to pre-clinical and/or clinical data suggesting an impact on beta cell function in those who have beta cells capable of C-peptide secretion.

TNFα is a pro-inflammatory cytokine that has important roles in the immunopathogenesis of a number of human autoimmune diseases, including T1D. TNFα may promote diabetes autoimmunity by enhancing the recruitment of inflammatory cells to the islets, activating cells and enhancing autoantigen presentation (27,28). TNF activates vascular endothelium, upregulating MHC I and adhesion molecules (29–31). In murine models of T1D, some of the first cells to infiltrate islets are dendritic cells. Dendritic cells and other antigen-presenting cells, which are critical for beta cell antigen presentation to T cells, are activated by TNFα by up regulation of MHC I and II and costimulatory molecules (32). TNFα also directly increases MHC I, and synergizes with interferon gamma to upregulate MHC II on beta cells, both of which appear to increase their susceptibility to T cell killing (reviewed in (33)). Rodent models of autoimmune diabetes show that antagonizing TNFα can delay, prevent or reverse disease whereas TNFα production in the pancreas can accelerate disease (34). TNFα also has direct cytostatic effects and impairs insulin production and secretion, and it has cytocidal activity, killing beta cells directly. Therefore, TNFα has potent metabolic effects that may contribute to T1D by increasing beta cell stress and death.

The clinical effect of TNF blockade on preservation of beta cell function was demonstrated in a pilot study using etanercept, an early-to-market anti-TNF drug. In this study of individuals with recently diagnosed T1D, etanercept-treated individuals had higher C-peptide AUC and lower HbA1c as compared to placebo control subjects (35). Eight of nine subjects (89%) in the anti-TNF treated group maintained or increased C-peptide secretion at 6 months post enrollment as compared with only 1/8 placebo treated subjects (13%). The preservation of insulin secretion seen in this pilot trial for etanercept is similar to that from the initial data for teplizumab (36), which has since moved into fully-powered trials in both the new-onset (37) and at-risk settings (completed enrollment, NCT01030861) and has been tested in subjects further from clinical diagnosis (38).

Golimumab (SIMPONI®) is a fully human monoclonal antibody which binds to TNFα with high affinity and specificity and neutralizes TNFα bioactivity. Golimumab is approved by the FDA for treatment of several arthritides and ulcerative colitis, and has been tested for use in juvenile idiopathic arthritis and pediatric ulcerative colitis. Based on the positive findings from the early etanercept trial, a phase 2 randomized, controlled clinical trial using golimumab to preserve beta cell function in newly diagnosed T1D (Stage 3) is currently being conducted (NCT02846545). Additionally, a Phase 1b study in subjects with Stage 2 T1D (multiple autoantibodies and abnormal glucose tolerance) is currently enrolling in Scandinavia (NCT03298542). Here, we propose to determine whether drugs in this class can improve beta cell function for those in Stage 4 T1D with little/no insulin secretion.

Glucagon-like peptide-1 (GLP-1) is an incretin hormone that is known to stimulate glucose-dependent insulin secretion. Furthermore, GLP-1 appears to have multiple positive effects on beta cells. It can regulate beta cell gene expression and may also play a role in increasing beta cell mass through regeneration, proliferation, and/or neogenesis. Data also suggest GLP may inhibit beta cell apoptosis (GLP-1 activity recently reviewed in (39)). There are multiple incretin mimetics available for clinical use, with differing mechanisms of action, PK, delivery methods, and costs. Broadly, these include DPP-IV inhibitors (GLP-1 is rapidly degraded by dipeptidyl peptidase-4, DPP-4) and GLP-1 receptor agonists. The differences between incretin mimetics may lead to differences in clinical effects on beta cells. Preclinical studies suggest that liraglutide (GLP-1R agonist), like exenatide (GLP-1R agonist), can increase beta cell mass, stimulate beta cell proliferation, increase beta cell neogenesis, and inhibit beta cell apoptosis (40,41). Liraglutide in combination with anti-IL21 reversed established disease in the NOD model (42) and this drug combination is currently under investigation in a trial to preserve beta cell function in adults with new onset T1D (NCT02443155). We are testing liraglutide due to intriguing clinical and pre-clinical data, but we recognize that data are inconsistent as to whether incretin mimetics have a significant impact on beta cell function in adults. For example, exenatide (GLP-1R agonist) has been shown to improve outcomes in islet transplant recipients (43); yet had no impact on beta cell function when tested in conjunction with daclizumab (anti IL2R Ab) in those with longstanding T1D (44). In this regard, it is important to note that, in contrast to the Rother study (44), our population includes those without C-peptide but with proinsulin and our outcome measure is different. Four weeks of liraglutide was also tested in C-peptide positive and negative subjects in a Danish cohort; investigators noted a reduction in insulin use for both groups. While the C-peptide positive group showed no change in insulin secretion after 1 month of liraglutide, follow-up measures of beta cell function were only performed in a subset of C-peptide positive subjects (45). Clinical trial data in type 2 diabetes reveal an improvement in beta cell function in patients treated with liraglutide, as assessed by homeostasis model assessment (HOMA)-B analysis (46,47) and proinsulin: insulin ratio (47,48). Additionally, liraglutide enhances first-and second-phase insulin secretion and restores beta cell sensitivity to glucose in clinical studies (46,47). It also improved glycemic control in a population with T1D, although beta cell function was not assessed (49). For these reasons, we hypothesize that liraglutide may induce transient *in vivo* functional changes to severely dysfunctional or de-differentiated beta cells in subjects with longstanding T1D and proinsulin presence.

# Study Objectives

## Primary Objective

To determine whether either of two therapeutic interventions can transiently improve beta cell function in individuals with severely dysfunctional or de-differentiated beta cells (MMTT C-peptide <0.017 pmol/mL and with detectable or undetectable proinsulin). This outcome will be assessed by increased C-peptide (primary outcome) or IAPP production, or a reduction in the PI/C or pro-IAPP/IAPP ratios.

## Secondary Objectives

1. To determine the relationship between impaired proinsulin processing with impaired processing of pro-IAPP in individuals with longstanding T1D
2. To determine if the effect on beta cell function is sustained for 2 to 4 weeks after cessation of therapy
3. To explore the relationship of metabolic measures to immunologic response to therapy.

## Safety Objective

Evaluate adverse effects associated with short-term administration of liraglutide or golimumab in T1D.

# Study Design

## Overview

This protocol involves two independent open label, proof of concept studies to determine whether therapeutic interventions can transiently improve beta cell function in those who do or do not secrete proinsulin and little/no C-peptide. This is a mechanistic study aiming to determine whether severely dysfunctional or de-differentiated beta cells can be induced to secrete C-peptide; it is not designed to evaluate the clinical impact of these interventions.

Each individual may participate in one or both of these independent studies with a period of 3 months between each study.


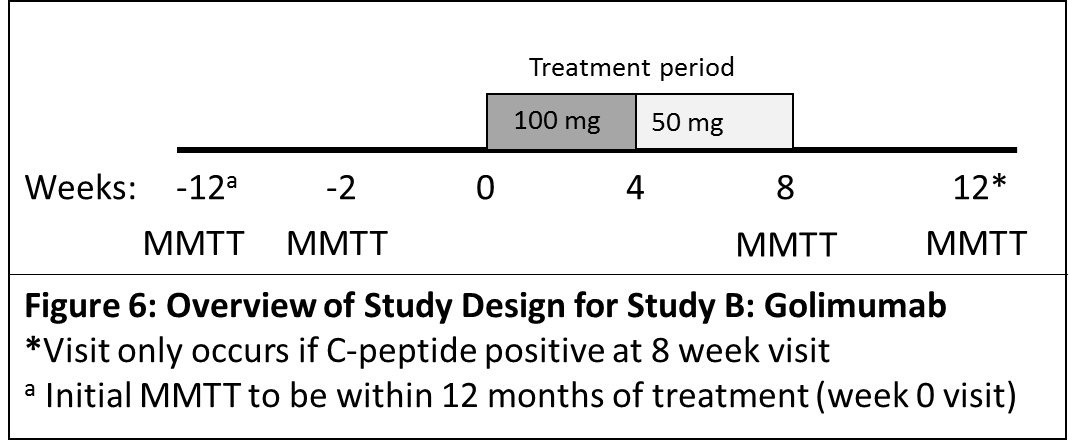

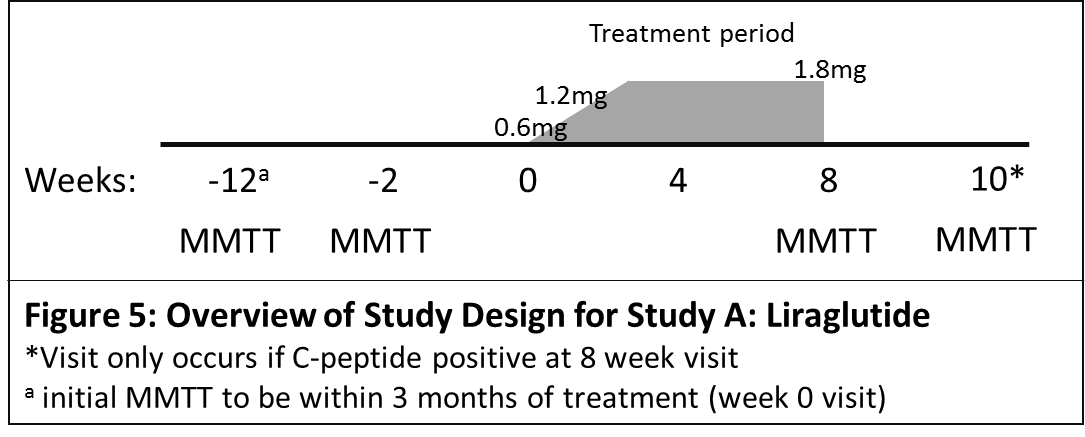
Subjects diagnosed with T1D who do or do not secrete proinsulin with little/no C-peptide and meeting other eligibility criteria will receive therapy for two months: Study A, liraglutide daily (*Figure 5*); Study B, golimumab every two weeks (*Figure 6*). Measures of beta cell function will be done before and after treatment, and if C-peptide is present at the end of therapy, follow-up evaluation will be done to assess duration of response.

## Endpoints

### Primary Endpoint

Proportion of individuals with peak MMTT stimulated C-peptide >0.017 pmol/mL at 8 weeks

### Secondary/Exploratory Endpoints

- Changes in proinsulin, proinsulin to C-peptide ratio, IAPP and pro-IAPP between screening and 8 week MMTT
  - The proinsulin assays will be performed in a batch at the end of study
- The proportion of those with C-peptide >0.017 pmol/mL at 8 weeks who are above and below that threshold after cessation of therapy (at 10 weeks for Study A: liraglutide and 12 weeks for Study B: golimumab)
- Exploratory: Relationship of metabolic measures to genetic, genomic, and immunological response

## Eligibility

### Inclusion criteria

1. ≥ 3 years from Type 1 diabetes diagnosis
2. Males and females 18-50 years of age, inclusive
3. Peak MMTT stimulated C-peptide <0.017 pmol/mL
4. Females of child-bearing potential must be willing to use effective birth control for 12 weeks
5. Willing and able to give informed consent for participation
6. HbA1c ≤ 8.5%

### Exclusion criteria

1. Concurrent use of non-insulin therapies aimed to control hyperglycemia or use within the past 30 days of screening MMTT (V-2).
2. History of severe reaction or anaphylaxis to human, humanized or murine monoclonal antibodies.
3. Diagnosis of liver disease or elevated hepatic enzymes, as defined by ALT or AST> 1.5 x the upper limit of age-determined normal (ULN) .
4. Females who are pregnant or lactating.
5. Receipt of an immune modulating biologic or investigational drug within 3 months or 5 half-lives before enrollment.
6. History of other clinically significant autoimmune disease needing chronic therapy with biologics or steroids with the exception of celiac and stable thyroid disease.
7. Current use of any medication known to significantly influence glucose tolerance (e.g. oral steroids, atypical antipsychotics, diphenylhydantoin, niacin).
8. Any medical or psychological condition that in the opinion of the principal investigator would interfere with the safe completion of the trial.
9. For Study A (liraglutide)
   1. Any history of pancreatitis or elevated amylase or lipase.
   2. Any personal or family history of thyroid C-cell tumors, including medullary thyroid carcinoma (MTC).
   3. Any personal or family history of multiple endocrine neoplasia syndrome type 2.
   4. Hypersensitivity to liraglutide.
   5. Previous treatment with liraglutide.
   6. Known history of clinically significant gastroparesis.
10. For Study B (golimumab)
    1. Any history of recent (within 3 months) serious bacterial, viral, fungal, or other opportunistic infections.
    2. Any history of demyelinating diseases (such as multiple sclerosis), heart failure, or left ventricular dysfunction.
    3. Serologic evidence of current or past HIV, Hepatitis B, or Hepatitis C.
    4. Positive QuantiFERON or PPD TB test, history of tuberculosis, or active TB infection.
    5. Active infection with EBV, defined by real-time PCR.
    6. Active infection with CMV, defined by real-time PCR.
    7. Any of the following hematologic abnormalities at screening:
       1. White blood count <3,000/μL or >14,000/μL
       2. Lymphocyte count <500/μL
       3. Platelet count <140,000 /μL
       4. Hemoglobin <8.5 g/dL
       5. Neutrophil count <2,000 cells/μL
    8. Receipt of live vaccine (in the 6 weeks before treatment)

## Study Duration

Enrollment in these two independent studies is anticipated to occur within 24 months. The rate of enrollment may vary as data is collected and evaluated.

## Study sites

The study will be conducted at two locations: Benaroya Research Institute in Seattle, WA and Rocky Mountain Diabetes Center, in Idaho Falls, ID.

# Study Procedures

## Screening Visits (visit numbers -2,-1)

Potentially eligible participants will undergo screening visit(s) to determine eligibility for the study.   Eligible individuals must have two consecutive MMTTs within a 12 month window from the treatment visit (V0) with peak C-peptide <0.017 pmol/mL. Any participant whose other laboratory values exclude the participant from the study may undergo repeat assessments.  In these cases, two subsequent tests are required to confirm eligibility.

## Administration of Study Drug

### Formulation

#### Liraglutide: (Victoza®; NovoNordisk).

This is an acylated human GLP-1 receptor agonist. It is FDA approved for the treatment of type 2 diabetes and for prophylaxis of major adverse cardiovascular events among people with type 2 diabetes. For this trial, the drug will be initially administered as 0.6 mg SC daily with increase to 1.2 mg/day at approximately 7 days and then to 1.8 mg/day approximately 7 days later as tolerated. Dose escalation will occur within a two week period.

#### Golimumab: (SIMPONI®; Janssen).

This is a human monoclonal antibody that binds to soluble and transmembrane human TNFα resulting in inhibition of TNFα activity by preventing binding of TNFα to receptors. It is an FDA approved therapy for ankylosing spondylitis, psoriatic arthritis, moderate to severe active rheumatoid arthritis in conjunction with methotrexate and moderate to severe active ulcerative colitis. Dosing for the ongoing trial in recent onset T1D (NCT02846545) will be used in this study. 100 mg golimumab SC will be administered at week 0 and 2, followed by 50 mg golimumab SC every two weeks from week 4 - 8.

### Preparation and Accountability

Golimumab and liraglutide for this study will be stored at the VMMC Investigational Pharmacy and in an equivalent location at Rocky Mountain Diabetes Center. Records of the disposition of the investigational product, including the date and quantity of drug that was received, the participants to whom drug was dispensed (by participant accounting), and an account of any drug accidentally or deliberately destroyed will be kept.

A drug administration log will be kept current for each participant and will contain the identification of each participant and the date and quantity of drug dispensed. All remaining unused investigational product will be returned to the Investigational Drug Services (IDS) at Virginia Mason and destroyed according to the standard operating procedures of the IDS.

### Initial Treatment Visit (visit number 0)

Criteria for dosing: Review of systems with directed exam, including vital signs and medical history, clinical laboratory test results, and pregnancy test (as appropriate) will be done at the dosing visit. Investigator will confirm that participant still meets all eligibility criteria prior to dosing.

For study A: Liraglutide will be initiated at a dose of 0.6 mg SC at the initial treatment visit. Individuals will be instructed on self-administration of study drug. They will be dispensed study drug for home administration with instructions to increase to 1.2 mg/day and then to 1.8 mg/day as tolerated over a two week period. There is an approximate two month treatment period.

For study B: Golimumab will be initiated at a dose of 100 mg SC at the initial treatment visit. Individuals will be instructed on self-administration of study drug. They will be dispensed study drug for home administration with instructions to dose at 100 mg at week 2, and then 50 mg at weeks 4,6, and 8 for a maximum of 5 doses over two months.

### Dosing Windows

All attempts should be made to dose on the scheduled target dosing day, however due to scheduling and other events this may not be possible.

**For study A – Liraglutide:** One dose per day. Missed treatment doses will not made up and participants will take their next dose according to the dosing schedule. If Visit 2 is unable to be conducted within the preferred window, additional study drug will be provided for up to an additional 7 days to assure individuals are on study drug at the time of the visit.

**For study B – Golimumab:** Weeks 2, 4, 6 and 8 study agent dosing should be on the target dosing day. If a dose is missed, it may be taken as long as there is a minimum of 7 days between doses. The aim of the dosing schedule is to maintain drug levels during the treatment phase of the study.

## Subsequent study visits (visits 1, 2)

Review of systems and directed exam including AE assessments will be done. Clinical and mechanistic samples will be obtained. Visit 2 is the primary outcome visit. Individuals will undergo an MMTT.

## Follow-up study visit (visit 3)

Individuals who have C-peptide >0.017 pmol/mL at 8 weeks will return for follow-up assessment off study drug (at 10 weeks for liraglutide and 12 weeks for golimumab). Review of systems and directed exam including AE assessments will be done. Clinical and mechanistic samples will be obtained. Individuals will undergo an MMTT.

## Visit windows

**Initial visit (V-2):** The initial visit must occur within 12 months of the treatment visit (V0). The initial visit may occur within or outside the study.

**Screening Visit (V-1):** The screening visit must occur between 14 ± 7 days of the treatment visit (V0).

**Treatment visit (V0):** The initial treatment must begin within 21 days from the screening visit (V-1) MMTT.

**Study Visits (V1 and V2):** within 7 days on either side of the target date. The aim is to have participants on study drug at the time of Visit 2 (V2).

For Study A (liraglutide), the last dose of study drug will be at Visit 2 (V2). If visit 2 (V2) is unable to occur within the preferred window due to scheduling or other events such as intercurrent illness, up to 2 weeks of additional study drug may be provided.

For Study B (Golimumab) the last dose of study drug must occur within 14 days of Visit 2 (V2). If visit 2 (V2) is unable to occur within the preferred window due to scheduling or other events such as intercurrent illness, one additional dose of study drug may be provided.

**Follow up visit (V3):** If a participant is C-peptide positive at study visit 2 (V2) then they will have a follow up visit that must occur within 7 days on either side of the target date.

Appendix 1 and 2 presents the schedule of events for this trial.

## Assessments

### General Assessments

- Informed consent: written informed consent will be obtained from the participant before any study assessments or procedures are performed
- Eligibility criteria: eligibility for study participation will be assessed during the screening period
- Medical History: Relevant medical history, including history of current and previous disease, and review of systems will be obtained. Adverse events will be assessed. A directed physical exam will be conducted as indicated by medical history
- Concomitant medications: concomitant medications and their indications will be recorded

### Clinical Laboratory Assessments

- Islet autoantibodies: Antibody levels will be measured for all subjects at screening unless evidence of one or more islet antibodies has been previously documented.
- Basic Metabolic Panel: Electrolytes (sodium, potassium, chloride, total CO2, calcium), glucose, blood urea nitrogen (BUN), creatinine, GFR.
- Liver tests (AST and ALT)
- Hematology: Includes RBC, hematocrit, hemoglobin, platelet count, WBC and differential,
- Pregnancy testing: Females with reproductive capacity will undergo a urine pregnancy test at screening and subsequent study visits
- For study A (liraglutide)
  - Serum amylase, lipase
  - Lipid panel: Total cholesterol, HDL, LDL, triglycerides
- For study B (golimumab)
  - Infectious disease serology: Serology will be performed at screening for HBV, HCV, HIV, EBV, CMV
  - Viral load testing: Viral load testing by PCR will be performed for CMV and EBV at screening
  - QuantiFERON or PPD TB test: Will be performed at screening

### Metabolic Assessments

- 2-hour mixed meal tolerance test with measurements of C-peptide, proinsulin, pro-IAPP and IAPP, and glucose
- HbA1c

### Mechanistic Assessments

#### Mechanistic samples

Samples will be drawn at each study visit which will become part of the Benaroya Research Institute’s Immune Mediated Disease Repository. As such, they will be used for genetic, genomic, and immunologic studies related to T1D and the mechanisms of action of the therapies being tested.

# PARTICIPANT SAFETY

### Benefit of Participation

This study will not provide any direct benefit to the study subjects. Future patients may benefit from these studies by virtue of knowledge gained about ability of severely dysfunctional beta cells to recover function.

### Risks of Participation and Mitigation of Risks

**Study procedures: Blood draws and Mixed Meal Tolerance Testing (MMTT)**

There is minimal risk as a result of study procedures such as blood draw and IV placement which may result in discomfort or bruising and rarely phlebitis.

**Study medications: Liraglutide**

Common side effects of liraglutide include gastrointestinal symptoms (43%) with nausea (18-20%), vomiting (6-9%), diarrhea (10-12%) or other GI discomfort, headache (10-11%), URI (7-11%), injection site reaction (2%) and hypoglycemia associated with concomitant use with insulin or insulin secretagogues. There are other very rare side effects (<1%) including anaphylaxis, and pancreatitis of uncertain relationship with study drug. Thyroid tumors have been seen in animal models.

Specific concerns and risk mitigation for Liraglutide:

- GI symptoms (43%): Nausea (18% to 20%), diarrhea (10% to 12%), decreased appetite (9% to 10%), dyspepsia (4 to 9%), and vomiting (6% to 9%).

The symptoms usually are dose-related, and may decrease in frequency/severity with gradual titration and continued use.

The GI side-effects will be mitigated by use of dose escalation: subjects will start liraglutide at 0.6 mg SC daily and then titrate up to 1.2 mg SC daily over approximately 7 days and eventually 1.8mg SC daily over a two week period. Individuals who cannot tolerate 1.8 mg SC daily will receive 1.2 mg SC daily.

- Hypoglycemia: Concomitant use of insulin with liraglutide may result in hypoglycemia. Insulin dose adjustments may be recommended by a qualified staff member during regular phone or text check-ins and/or at clinic visits at Clinical Research Center.
- Hypersensitivity reactions (<1%): Serious hypersensitivity reactions, including anaphylactic reactions and angioedema, have been reported with use; permanently discontinue therapy in the event of a hypersensitivity reaction.
- Pancreatitis (<1%): Increased serum lipase (8%) and amylase (1%). Cases of acute and chronic pancreatitis have been reported; monitor for signs and symptoms of pancreatitis (eg, persistent severe abdominal pain which may radiate to the back and which may or may not be accompanied by vomiting). If pancreatitis is suspected, discontinue use of the medication and seek immediate medical attention.
- Gallbladder disease (1 to 2%): Use of GLP-1 agonists may increase risk of gallbladder and bile duct disease. Cholelithiasis (2%) and cholecystitis (1%) have been reported in patients treated with liraglutide; gallbladder studies and further clinical assessment are indicated if cholelithiasis is suspected.
- Thyroid tumors: Dose-dependent and treatment duration-dependent thyroid C-cell tumors have developed in animal studies with liraglutide therapy; however, it is unknown whether liraglutide will cause thyroid C-cell tumors, including MTC, in humans. Participants will be counseled on the potential risk of MTC with the use of liraglutide and informed of symptoms of thyroid tumors (eg, neck mass, dysphagia, dyspnea, persistent hoarseness). Individuals with a personal or a family history of MTC and individuals with multiple endocrine neoplasia syndrome type 2 (MEN2) will be excluded in the study. The risk of developing thyroid tumor is very minimal due to the short duration of study medication (8 weeks).

**Study medications: Golimumab**

Common side effects of Golimumab include viral (4-5%), fungal (<2%) and bacterial (1%) infections, hypertension (3%), increase in liver function (increase serum ALT 2-8%; increase serum AST <5%), dizziness (<2%) and paresthesia (<2%), decrease in neutrophils (<5%) and leukopenia (1%), as well as injection site reaction (3-6%). Serious side effects (<1%) include hypersensitivity, disseminated infections, demyelinating disease, and congestive heart failure (CHF). Lymphoma and other malignancies associated with TNF–blocking agents have been reported.

Specific concerns and risk mitigation for Golimumab:

- Serious infection (<1%): Patients treated with golimumab are at an increased risk of developing serious infections including active tuberculosis, invasive fungal infections, bacterial, viral and other opportunistic pathogens. Subjects will be monitored closely for signs/symptoms of infection. Medication will be discontinued in the event of serious infection or sepsis. Individuals will be excluded who have either current infections or evidence of past infections including TB, HIV, and hepatitis.
- Demyelinating disease (<1%): Rare cases of new-onset or exacerbation of demyelinating disorders (eg, multiple sclerosis, optic neuritis, Guillain-Barré syndrome, polyneuropathy) have been reported. The medication will be discontinued in participants who develop peripheral or CNS demyelinating disorders during treatment. Individuals with pre-existing or recent onset central or peripheral nervous system demyelinating disorders will be excluded in the study.
- CHF (<1%): Worsening and new-onset heart failure have been reported with golimumab and other TNF-blockers. Subjects will be monitored closely and medication discontinued with onset or worsening of symptoms. Individuals with CHF or decreased left ventricular function will be excluded from the study.
- Hematologic effects (1-5%): Pancytopenia, leukopenia, neutropenia, and thrombocytopenia have occurred with golimumab. If hematologic abnormalities occur that are grade 2 or greater, study medication may be discontinued or the lab may be repeated at the discretion of the investigator. If confirmed abnormal, study medication will be stopped. Individuals with underlying hematologic disorders will be excluded from the study.
- Liver function effects: an increase in serum ALT (2-8%), or an increase serum AST (<5%). If ALT or AST is increased to ≥2X ULN, study medication may be discontinued or the lab may be repeated at the discretion of the investigator. If confirmed abnormal to be ≥2X ULN, study medication will be stopped. .
- Hypersensitivity reactions (<1%): Severe systemic hypersensitivity reactions (including anaphylaxis) have been reported following subcutaneous administration. The medication will be discontinued immediately if symptoms and signs develop and appropriate treatment will be initiated.
- Malignancy: Lymphoma and other malignancies have been reported in patients receiving TNF-blocking agents. However, the impact of golimumab on the development and course of malignancy is not fully defined. Treatment with golimumab in this study will only be for 8 weeks, and thus risks associated with long-term treatment will be very unlikely to occur.

# Adverse Event Reporting and Documentation

## Overview

As the sponsor of the Study, the Sponsor Investigator shall be solely responsible for complying, within the required timelines, with any safety reporting obligation to competent Health Authorities, IRB/ECs and any participating co- or sub-investigators, as defined in applicable laws and regulations.

Safety will be monitored by the Clinical Investigator(s). Participant concerns and symptoms will be formally assessed at each visit and the study team in contact in between visits as clinically indicated.

## Definitions

### Adverse Event (AE)

An adverse event is any untoward medical occurrence in a clinical study subject administered a medicinal (investigational or non-investigational) product. An adverse event does not necessarily have a causal relationship with the treatment. An adverse event can therefore be any unfavorable and unintended sign (including an abnormal finding), symptom, or disease temporally associated with the use of a medicinal (investigational or non- investigational) product, whether or not related to that medicinal (investigational or non-investigational) product. (Definition per International Conference on Harmonisation [ICH]). This includes any occurrence that is new in onset or aggravated in severity or frequency from the baseline condition, or abnormal results of diagnostic procedures, including laboratory test abnormalities.

### Adverse Reaction

An adverse reaction means any adverse event caused by a drug. Adverse reactions are a subset of all suspected adverse reactions for which there is reason to conclude that the drug caused the event. Suspected adverse reaction means any adverse event for which there is a reasonable possibility that the drug caused the adverse event. For the purposes of safety reporting, “reasonable possibility” means there is evidence to suggest a causal relationship between the drug and the adverse event. A suspected adverse reaction implies a lesser degree of certainty about causality than an adverse reaction, which means any adverse event caused by a drug. Examples of evidence that suggest a causal relationship (reasonable possibility) between the drug and the adverse event include:

- A single occurrence of an event that is uncommon and known to be strongly associated with drug exposure.
- One or more occurrences of an event that is not commonly associated with drug exposure, but is otherwise uncommon in the populations exposed to the drug.

### Serious Adverse Event (SAE)

A serious adverse event (SAE) or reaction is defined as “any adverse event occurring at any dose that suggests a significant hazard, contraindication, side effect, or precaution.” An adverse event or suspected adverse reaction is considered “serious” if, in the view of the sponsor investigator, it results in any of the following outcomes:

- Death. A death that occurs during the study or that comes to the attention of the investigator during the protocol-defined follow-up after the completion of therapy must be reported whether it is considered to be treatment related or not;
- A life-threatening adverse event. A life-threatening event is any adverse therapy experience that, in the view of the investigator, places the participant at immediate risk of death from the reaction as it occurred;
- Inpatient hospitalization or prolongation of existing hospitalization with the exception of hospitalization relating to glycemic control in type 1 diabetes;
- Persistent or significant incapacity or substantial disruption of the ability to conduct normal life functions;
- Congenital anomaly or birth defect;
- Important medical events* that may not result in death, be life-threatening, or require hospitalization may be considered serious when, based upon appropriate medical judgment, they may jeopardize the patient or subject and may require medical or surgical intervention to prevent one of the outcomes listed above;
- Is a suspected transmission of any infectious agent via a medicinal product

*Medical and scientific judgment should be exercised in deciding whether expedited reporting is also appropriate in other situations, such as important medical events that may not be immediately life threatening or result in death or hospitalization but may jeopardize the subject or may require intervention to prevent one of the other outcomes listed in the definition above. These should usually be considered serious.

### Unexpected or Unlisted Adverse Event

An adverse event/reaction is considered unexpected when the nature (specificity) or severity of the event is not consistent with the risks described in the applicable product information, Investigator’s Brochure or the informed consent document. Unexpected refers to an experience that has not been previously observed. This includes events that occur more frequently than expected.

### Definitions Specific for Study B: Golimumab (SIMPONI®)

#### Adverse Events of Special Interest for Golimumab (SIMPONI®)

Adverse events of Special Interest include events that Janssen Scientific Affairs is actively monitoring as a result of previously identified signals detailed in the golimumab (SIMPONI®) prescribing information. These adverse events are:

- All malignancies

#### Individual Case Safety Report (ICSR) for Golimumab (SIMPONI®)

All reports of serious adverse events must contain the elements described for Individual Case Safety Reports (ICSR). A valid ICSR must contain the four minimum criteria required to meet regulatory reporting requirements.

- an identifiable subject (but not disclosing personal information such as the subject’s name, initials or address)
- an identifiable reporter (investigational site)
- a Janssen medicinal product
- an adverse event, outcome, or certain special situations

The minimum information required is:

- suspected Janssen medicinal product (doses, indication)
- date of therapy (start and end date, if available)
- batch or lot number, if available
- subject details (subject ID and country)
- gender
- age at AE onset
- reporter ID
- Janssen protocol ID
- adverse event detail (AE verbatim in English), onset date, relatedness, causality, action taken, outcome, (if available)

#### Product Quality Complaint (PQC) for Golimumab (SIMPONI®)

A product quality compliant is defined as any suspicion of a product defect related to a potential quality issue during manufacturing, packaging, release testing, stability monitoring, dose preparation, storage or distribution of the product, or delivery system. Not all PQCs involve a subject. Lot and batch numbers are of high significance and need to be collected whenever available.

Examples of PQC include but not limited to:

- Functional Problem: e.g., altered delivery rate in a controlled release product
- Physical Defect: e.g. abnormal odor, broken or crushed tablets/capsules
- Potential Dosing Device Malfunction: e.g., auto injector button not working, needle detaching from syringe
- Suspected Contamination
- Suspected Counterfeit

## Safety Assessments and Collection of Safety Data

This is an exploratory study designed to assess the effects of two months of therapy on β cell function. All adverse events, regardless of causality, special reporting situations, and product quality complaints will be collected from the time a subject has signed and dated an Informed Consent Form (ICF) until at least 30 days after the last dose (which may include contact for follow-up safety).

The Investigator(s) will treat participants with adverse events appropriately and observe them at suitable intervals until the events resolve or stabilize. Adverse events may be discovered through:

- observation of the participant;
- questioning the participant;
- unsolicited complaints by the participant.

All serious adverse events, adverse events of special interest, product quality complaints, and special situations including pregnancies, whether serious or non-serious, related or not related, following exposure to study drug will be documented by the Sponsor Investigator (or sub-Investigators) and recorded and maintained in subject’s source documents. Serious and Unexpected adverse events deemed possibly or probably drug related will be reported to the IRB per requirements within 24 hours of notification of the event. Events will be assessed and reported consistent with the ICH Guideline for Good Clinical Practice, 21 CFR 312.32 for expedited safety reporting, per the guidance of the DHHS Office for Human Research Protections (OHRP), and according to any reporting requirements outlined in this protocol.

Events with the exception of hypoglycemia or hyperglycemia will be graded per the National Cancer Institute’s Common Terminology Criteria for Adverse Events Version 5.0 (published November 27, 2017). An adverse hypoglycemic event is defined as one resulting in loss of consciousness, seizure, or requiring assistance of others due to altered state of consciousness. An adverse hyperglycemic event is one resulting in diabetic ketoacidosis (DKA). The Clinical Investigator(s) will treat participants with adverse events appropriately and observe them at suitable intervals until the events resolve or stabilize. Adverse events may be discovered through:

Duration (start and stop dates and times), severity/grade, outcome, treatment and relationship to study drug (not, unlikely, possibly, probably, or definitely related) will be recorded on source documents. The batch and lot number of the product will be recorded for all patients, including dose and date of infusion.

### Procedures for Reporting to Janssen Scientific Affairs for Golimumab (SIMPONI®)

The Sponsor Investigator will provide safety information to Janssen Scientific Affairs on all adverse events for Janssen Medicinal Products regardless of causality and special situations excluding those from subjects not exposed to a Janssen Medicinal Product and product quality complaints with or without an adverse event as described in section 8.2.5.3.

For the purposes of this study, the Janssen medicinal product is: SIMPONI® (golimumab).

### SAEs and Special Reporting Situations

All serious adverse events that have not resolved by the end of the study, or that have not resolved upon discontinuation of the subject’s participation in the study, must be followed until any of the following occurs:

- The event resolves;
- The event stabilizes;
- The event returns to baseline, if a baseline value/status is available;
- The event can be attributed to agents other than the study drug or to factors unrelated to study conduct; or

It becomes unlikely that any additional information can be obtained (subject or health care practitioner refusal to provide additional information, lost to follow-up after demonstration of due diligence with follow-up efforts.

#### Additional SAE Reporting requirements for Golimumab (SIMPONI®)

In addition to the above, serious adverse events as defined in Section 8.2.3 will be reported to Janssen Scientific Affairs using FDA Form MedWatch 3500A within 24 hours of becoming aware of the event. Serious adverse events will be reported for 30 days after the last dose of study drug.

All follow-up information for serious adverse events that are not resolved at the end of the study or by the time of patient withdrawal must be reported directly by the Sponsor Investigator, within 24 hours of becoming aware, to Janssen Scientific Affairs.

All available clinical information relevant to the evaluation of a related SAE, adverse events of special interest or special situation is required. The Sponsor Investigator is responsible for ensuring that these cases are complete and if not are promptly followed-up. A safety report is not considered complete until all clinical details needed to interpret the case are received. Reporting of follow-up information should follow the same time line as initial reports and contain the elements summarized in section 8.2.5.2.

#### Additional Special Reporting Situations for Golimumab (SIMPONI®)

In addition to the above, special reporting situations that occur during the study period, as defined below, will be reported to Janssen Scientific Affairs using the MedWatch 3500A Form within 24 hours of becoming aware of the event. Safety information to be included in each safety report is summarized below.

Adverse events of interest for a Janssen medicinal product that require expedited reporting and/or safety valuation include, but are not limited to:

- Drug exposure during pregnancy (maternal and paternal);
- Overdose of a Janssen medicinal product;
- Exposure to a Janssen medicinal product from breastfeeding;
- Suspected abuse/misuse of a Janssen medicinal product;
- Inadvertent or accidental exposure to a Janssen medicinal product;
- Medication error involving a Janssen medicinal product (with or without patient exposure to the Janssen medicinal product, e.g., name confusion);
- Suspected transmission of any infectious agent via administration of a medicinal product;
- Unexpected therapeutic or clinical benefit from use of a Janssen medicinal product

These safety events may not meet the definition of an adverse event; however, from a Janssen Scientific Affairs perspective, they are treated in the same manner as adverse events. Any special situation that meets the criteria of a serious adverse event should be recorded on a Serious Adverse Event Report Form and be reported to the Janssen Scientific Affairs within 24 hours of becoming aware of the event.

#### Adverse Events of Special Interest for Golimumab (SIMPONI®)

Adverse events of special interest, as defined in Section 8.2.5.1, will be reported to Janssen Scientific Affairs within 24 hours of learning of the event.

#### Product Quality Complaints (PQC) for Golimumab (SIMPONI®)

A PQC may have an impact on the safety and efficacy of the product. Timely, accurate, and complete reporting and analysis of PQC information from studies are crucial for the protection of patients, investigators, and Janssen Scientific Affairs, and are mandated by regulatory agencies worldwide. Janssen Scientific Affairs has established procedures in conformity with regulatory requirements worldwide to ensure appropriate reporting of PQC information. Lot and/or Batch #s shall be collected or any reports failure of expected pharmacological action (i.e., lack of effect). The product should be quarantined immediately and if possible, take a picture.

All initial PQCs involving a Janssen medicinal product under study must be reported to Janssen Scientific Affairs by the Sponsor Investigator within 24 hours after being made aware of the event. The Janssen contact will provide additional information/form to be completed.

If the defect for a Janssen medicinal product under study is combined with either a serious adverse event or non-serious adverse event, the Sponsor Investigator must report the PQC to Janssen Scientific Affairs according to the serious adverse event reporting timelines. A sample of the suspected product should be maintained for further investigation if requested by Janssen Scientific Affairs.

### Reporting pregnancy

Any subject who becomes pregnant during the study must be promptly withdrawn from the study and discontinue further participation.

The investigator should be informed immediately of any pregnancy in the participant or a partner pregnancy of a male participant occurring during the treatment period with either liraglutide or golimumab. The investigator should be available to counsel the participant or refer the participant (or partner) for counseling to discuss possible risks to the pregnancy and fetus. Pregnancy, pregnancy complication or pregnancy termination would be recorded as an AE. The pregnancy outcome will be recorded.

#### Reporting Pregnancy for Golimumab (SIMPONI®)

In addition to above, pregnancies in female participants or partners of male participants will be reported to Janssen Scientific Affairs within 24 hours of learning of the event using the FDA MedWatch 3500 A form. Follow-up information regarding the outcome of the pregnancy and any postnatal sequelae in the infant will be required. Depending on local legislation this may require prior consent of the partner of the male participant.

Any abnormal pregnancy outcomes (e.g., spontaneous abortions, fetal demise, stillbirths, and congenital anomalies) will be reported to Janssen as SAE within 24 hours of knowledge of the event. Safety information to be included in each safety report is summarized in section 8.2.5.2.

# Statistical Analysis Plan

## Endpoints:

### Primary Endpoint

The primary endpoint is the proportion of individuals with peak MMTT stimulated C-peptide >0.017 pmol/mL at 8 weeks.

### Secondary and Exploratory Endpoints

Secondary endpoints include changes in proinsulin, proinsulin to C-peptide ratio, IAPP and pro-IAPP between screening and 8 week MMTT. The duration of effect will be determined by assessing the proportion of those with C-peptide >0.017 pmol/mL at 8 weeks who are above and below that threshold after cessation of therapy (at 10 weeks for liraglutide and 12 weeks for golimumab).

Exploratory endpoints will include the relationship between the metabolic effects of therapy and future genetic, genomic, and immunologic studies using stored samples

## Sample size:

These trials are pilot, mechanistic, proof of concept studies to determine whether severely dysfunctional or de-differentiated beta cells can be induced to secrete C-peptide. As a proof of concept study, we wanted to detect such an outcome in even a small proportion of individuals. Thus our study is powered to detect a positive outcome (defined as the presence of MMTT stimulated C-peptide >0.017 pmol/mL at 8 weeks) in at least 20% of participants for each study. Studying 15 participants gives 83% power to detect a positive outcome in 20% of participants, at an alpha of 0.05. This number of participants would also give 90% power to detect a larger proportion of individuals with a positive outcome (25-30% of subjects) at the same alpha.

Individuals unable to tolerate or who are non-compliant with study medication through 8 weeks or who do not undergo week 8 MMTT will be replaced. It is important to note that this is unlikely as the study medications are widely used clinically, yet if this occurs in 50% of more of the first 10 subjects for each of the trials, the study may be paused or stopped and feasibility re-evaluated.

# ETHICAL CONSIDERATIONS AND COMPLIANCE WITH GOOD CLINICAL PRACTICE

## Statement of Compliance

This study will be conducted at two clinical sites; Benaroya Research Institute in Seattle, WA and Rocky Mountain Diabetes Center in Idaho Falls, ID. The study will be conducted in compliance with the protocol and consistent with current Good Clinical Practices (GCP), adopting the principles of the Declaration of Helsinki, and all applicable regulatory requirements (ICH E6, 45CFR46, and FDA 21CFR sections 11, 50, 56, 312).

Prior to study initiation, the protocol and the informed consent documents will be reviewed and approved by the IRBs providing oversite for the two participating institutions: Benaroya Research Institute at Virginia Mason Institutional Review Board (IRB) for the Benaroya Research Institute clinical site and Western IRB (WIRB) for Rocky Mountain Diabetes Center clinical site. Any amendments to the protocol or consent materials will also be approved by these IRBs.

## Informed Consent

The process of assuring that individuals are making an informed decision about participating in this study includes both verbal and written communication. All participants must read, sign, and date a consent form before participating in the study, taking the study drug, and/or undergoing any study-specific procedures.

The informed consent form must be updated or revised whenever important new safety information is available, whenever the protocol is amended, and/or whenever any new information becomes available that may affect participation in the trial.

A copy of the informed consent will be given to a prospective participant for review.

## Withdrawal of Subjects from the Study

A subject may be withdrawn from the study at any time if the subject, the investigator, or the Sponsor feels that it is not in the subject’s best interest to continue.

All subjects are free to withdraw from participation at any time, for any reason, specified or unspecified, and without prejudice.

Reasonable attempts will be made by the investigator to provide a reason for subject withdrawals.  The reason for the subject’s withdrawal from the study will be specified in the subject’s source documents.

For study A (liraglutide), individuals unable to tolerate at least 1.2 mg/daily by two weeks after the initial treatment visit, or those for either study who are subsequently known to be non-compliant or unable to tolerate 2 months of study medication, will be withdrawn from study and will be replaced. All subjects who have received any study medication will be considered in the safety evaluation cohorts. Those with adverse events possibly related to study medication will be followed as clinically appropriate until resolution.

## Privacy and Confidentiality

A participant’s privacy and confidentiality will be respected throughout the study. Each participant will be assigned a sequential identification number. This number, rather than the participant’s name, will be used in data analysis and reports

# Data Collection, Monitoring, and Sample Retention

## Data Collection Instruments

The Clinical Investigators will prepare and maintain adequate and accurate source documents designed to record all observations and other pertinent data for each subject treated with the study drug. Data from source documents will be entered into protocol specific case report forms. The Investigator is responsible for all information collected on subjects enrolled in this study.

## Archival of Data

The database is safeguarded against unauthorized access by established security procedures; appropriate backup copies of the database and related software files will be maintained.  Databases are backed up by the database administrator in conjunction with any updates or changes to the database.

## Monitoring

This study will be monitored by representatives of the Benaroya Research Institute Clinical Research Program Administrative Office according to the U.S. CFR Title 21 Parts 50, 56, and 312 and ICH Guidelines for GCP (E6).

All study documents (patient files, signed informed consent forms, copies of CRFs, Study File Notebook, etc.) will be available for monitors and maintained for at least two years after the study is completed.

## Sample Retention

Specimens collected in this trial will be deposited into the BRI sample repository and may be used to evaluate additional responses as new research tools become available or exploratory hypotheses are generated. Residual specimens may be used by investigators at BRI or at collaborating institutions for development of new assays or for comparisons across studies.

# Schedule of assessments

## Study A: Schedule of Assessments for Liraglutide

| Visit number (week) | -2^1^ | -1 | 0 | 1 | 2 | 3^2,3^ |
| --- | --- | --- | --- | --- | --- | --- |
|  | Initial MMTT | Screening visit | Initial treatment visit | Week 4 | Primary Outcome visit | Follow-up |
| Time point (day) | Within 3 months of visit 0 | -14±7 days | 0 | 28±7 days | 56 ±7 days | 70 ± 7 days |
| Study medication |  |  | x | x | x |  |
| General assessments (initial and interim history and directed physical) |  | x | x | x | x | x |
| Adverse events assessment* |  |  | x | x | x | x |
| MMTT | x | x |  |  | x | x |
| Islet autoantibodies^6^ | x |  |  |  |  |  |
| HbA1c | x | x |  |  | X | x |
| Concomitant medications including insulin dose* | x | x | x | x | x | x |
| Urine Pregnancy Test^5^ | x | x | x | x | x | x |
| Electrolytes, creatinine, BUN |  | X |  |  |  |  |
| Liver function tests |  | x^4^ |  |  |  |  |
| Amylase, Lipase |  | x^4^ |  |  |  |  |
| CBC with differential  and platelet count |  | x^4^ |  | X | X | X |
| Samples for mechanistic studies | x | x | x | x | x | x |

*Weekly contacts with all participants, during treatment period, will be done to query for adverse events and compliance with study medication.

1. Initial MMTT may be done within 3 months of initial treatment visit
2. Visit 3 only if MMTT C-peptide at visit 2 >0.017 pmol/mL;
3. Any adverse events or abnormal clinical laboratory values possibly related to study medication will be followed off study medication up to visit 3 or as appropriate until resolved.
4. Screening lab results may be obtained at Initial MMTT (visit -2) if scheduled within 30 days of Initial Treatment Visit.
5. Females with reproductive capacity only.
6. Islet autoantibodies testing to be performed only on participants that do not have documented results from previous testing.

## Study B: Schedule of Assessments for Golimumab:

| Visit number (week) | -2^1^ | -1 | 0 | 1 | 2 | 3^2,3^ |
| --- | --- | --- | --- | --- | --- | --- |
|  | Initial MMTT | Screening visit | Initial treatment visit | Week 4 | Primary Outcome visit | Follow-up |
| Time point (day) | Within 12 months of visit 0 | -14±7 days | 0 | 28±7 days | 56 ±7 days | 84 ± 7 days |
| Study medication |  |  | x | x | x |  |
| General assessments (initial and interim history and directed physical) |  | x | x | x | x | x |
| Adverse events assessment* |  |  | x | x | x | x |
| MMTT | x | x |  |  | x | x |
| Islet autoantibodies^6^ | x |  |  |  |  |  |
| HbA1c | x | x |  |  | X | x |
| Concomitant medications including insulin dose* | x | x | x | x | x | x |
| HIV, HBV, HCV serology |  | x^4^ |  |  |  |  |
| QuantiFERON TB test |  | x^4^ |  |  |  |  |
| EBV, CMV Serology |  | x^4^ |  |  |  |  |
| EBV, CMV viral load |  | x^4^ |  |  |  |  |
| Urine Pregnancy Test^5^ | x | x | x | x | x | x |
| Basic Metabolic Panel |  | X |  |  |  |  |
| Liver tests (AST,ALT) |  | x^4^ |  | x | x |  |
| CBC with differential  and platelet count |  | x^4^ |  | x | x |  |
| Samples for mechanistic studies | x | x | x | x | x | x |

*Weekly contacts with all participants, during treatment period, will be done to query for adverse events and compliance with study medication.

1. Initial MMTT may be done within 12 months of initial treatment visit
2. Visit 3 only if MMTT C-peptide at visit 2 >0.017 pmol/mL;
3. Any adverse events or abnormal clinical laboratory values possibly related to study medication will be followed off study medication up to visit 3 or as appropriate until resolved.
4. Screening lab results may be obtained at Initial MMTT (visit -2) if scheduled within 30 days of Initial Treatment Visit.
5. Females with reproductive capacity only.
6. Islet autoantibodies testing to be performed only on participants that do not have documented results from previous testing.

# References

1. Mayer-Davis EJ, Lawrence JM, Dabelea D, Divers J, Isom S, Dolan L, et al. Incidence Trends of Type 1 and Type 2 Diabetes among Youths, 2002–2012. N Engl J Med [Internet]. 2017;376(15):1419–29. Available from: http://www.nejm.org/doi/10.1056/NEJMoa1610187

2. Miller KM, Foster NC, Beck RW, Bergensta RM, DuBose SN, DiMeglio LA, et al. Current state of type 1 diabetes treatment in the U.S.: Updated data from the t1d exchange clinic registry. Diabetes Care. 2015;38(6):971–8.

3. Gagnum V, Saeed M, Stene LC, Leivestad T, Joner G, Skrivarhaug T. Low incidence of end-stage renal disease in childhood-onset type 1 diabetes followed for up to 42 years. Diabetes Care. 2018;41(3):420–5.

4. Lind M, Svensson A-M, Kosiborod M, Gudbjörnsdottir S, Pivodic A, Wedel H, et al. Glycemic Control and Excess Mortality in Type 1 Diabetes. N Engl J Med [Internet]. 2014;371(21):1972–82. Available from: http://www.nejm.org/doi/10.1056/NEJMoa1408214

5. Ali MK, Bullard KM, Saaddine JB, Cowie CC, Imperatore G, Gregg EW. Achievement of Goals in U.S. Diabetes Care, 1999–2010. N Engl J Med [Internet]. 2013;368(17):1613–24. Available from: http://www.nejm.org/doi/10.1056/NEJMsa1213829

6. Herold KC, Gitelman SE, Masharani U, Hagopian W, Bisikirska B, Donaldson D, et al. A single course of anti-CD3 monoclonal antibody hOKT3gamma1(Ala-Ala) results in improvement in C-peptide responses and clinical parameters for at least 2 years after onset of type 1 diabetes. Diabetes. 2005 Jun;54(6):1763–9.

7. Rigby MR, DiMeglio LA, Rendell MS, Felner EI, Dostou JM, Gitelman SE, et al. Targeting of memory T cells with alefacept in new-onset type 1 diabetes (T1DAL study): 12 month results of a randomised, double-blind, placebo-controlled phase 2 trial. lancet Diabetes Endocrinol. 2013 Dec;1(4):284–94.

8. Pescovitz MD, Greenbaum CJ, Krause-Steinrauf H, Becker DJ, Gitelman SE, Goland R, et al. Rituximab, B-lymphocyte depletion, and preservation of beta-cell function. N Engl J Med. 2009 Nov;361(22):2143–52.

9. Orban T, Bundy B, Becker DJ, DiMeglio LA, Gitelman SE, Goland R, et al. Co-stimulation modulation with abatacept in patients with recent-onset type 1 diabetes: a randomised, double-blind, placebo-controlled trial. Lancet. 2011 Jul;378(9789):412–9.

10. Rodriguez-Calvo T, Zapardiel-Gonzalo J, Amirian N, Castillo E, Lajevardi Y, Krogvold L, et al. Increase in Pancreatic Proinsulin and Preservation of β-Cell Mass in Autoantibody-Positive Donors Prior to Type 1 Diabetes Onset. Diabetes. 2017 May;66(5):1334–45.

11. Piemonti L, Maffi P, Monti L, Lampasona V, Perseghin G, Magistretti P, et al. Beta cell function during rapamycin monotherapy in long-term type 1 diabetes. Diabetologia. 2011;54(2):433–9.

12. Eizirik DL, Miani M, Cardozo AK. Signalling danger: endoplasmic reticulum stress and the unfolded protein response in pancreatic islet inflammation. Diabetologia. 2013 Feb;56(2):234–41.

13. Furuta M, Carroll R, Martin S, Swift HH, Ravazzola M, Orci L, et al. Incomplete processing of proinsulin to insulin accompanied by elevation of Des-31,32 proinsulin intermediates in islets of mice lacking active PC2. J Biol Chem. 1998 Feb;273(6):3431–7.

14. Zhu X, Orci L, Carroll R, Norrbom C, Ravazzola M, Steiner DF. Severe block in processing of proinsulin to insulin accompanied by elevation of des-64,65 proinsulin intermediates in islets of mice lacking prohormone convertase 1/3. Proc Natl Acad Sci. 2002 Aug;99(16):10299–304.

15. Ward WK, LaCava EC, Paquette TL, Beard JC, Wallum BJ, Porte D. Disproportionate elevation of immunoreactive proinsulin in type 2 (non-insulin-dependent) diabetes mellitus and in experimental insulin resistance. Diabetologia. 1987 Sep;30(9):698–702.

16. Klimek AM, Soukhatcheva G, Thompson DM, Warnock GL, Salehi M, Rilo H, et al. Impaired Proinsulin Processing is a Characteristic of Transplanted Islets. Am J Transplant. 2009 Sep;9(9):2119–25.

17. Watkins RA, Evans-Molina C, Terrell JK, Day KH, Guindon L, Restrepo IA, et al. Proinsulin and heat shock protein 90 as biomarkers of beta-cell stress in the early period after onset of type 1 diabetes. Transl Res. 2016 Feb;168:96–106.e1.

18. Truyen I, De Pauw P, Jørgensen PN, Van Schravendijk C, Ubani O, Decochez K, et al. Proinsulin levels and the proinsulin:c-peptide ratio complement autoantibody measurement for predicting type 1 diabetes. Diabetologia. 2005 Nov;48(11):2322–9.

19. Røder ME, Knip M, Hartling SG, Karjalainen J, Akerblom HK, Binder C. Disproportionately elevated proinsulin levels precede the onset of insulin-dependent diabetes mellitus in siblings with low first phase insulin responses. The Childhood Diabetes in Finland Study Group. J Clin Endocrinol Metab. 1994 Dec;79(6):1570–5.

20. Sims EK, Chaudhry Z, Watkins R, Syed F, Blum J, Ouyang F, et al. Elevations in the Fasting Serum Proinsulin–to–C-Peptide Ratio Precede the Onset of Type 1 Diabetes. Diabetes Care. 2016 Sep;39(9):1519–26.

21. Snorgaard O, Hartling SG, Binder C. Proinsulin and C-peptide at onset and during 12 months cyclosporin treatment of type 1 (insulin-dependent) diabetes mellitus. Diabetologia. 1990 Jan;33(1):36–42.

22. Larsen CM, Faulenbach M, Vaag A, Vølund A, Ehses JA, Seifert B, et al. Interleukin-1-receptor antagonist in type 2 diabetes mellitus. N Engl J Med. 2007 Apr;356(15):1517–26.

23. Inge TH, Prigeon RL, Elder DA, Jenkins TM, Cohen RM, Xanthakos SA, et al. Insulin Sensitivity and β-Cell Function Improve after Gastric Bypass in Severely Obese Adolescents. J Pediatr. 2015 Nov;167(5):1042–1048.e1.

24. Wang J, Xu J, Finnerty J, Furuta M, Steiner DF, Verchere CB. The prohormone convertase enzyme 2 (PC2) is essential for processing pro-islet amyloid polypeptide at the NH2-terminal cleavage site. Diabetes. 2001 Mar;50(3):534–9.

25. Marzban L, Trigo-Gonzalez G, Zhu X, Rhodes CJ, Halban PA, Steiner DF, et al. Role of beta-cell prohormone convertase (PC)1/3 in processing of pro-islet amyloid polypeptide. Diabetes. 2004 Jan;53(1):141–8.

26. Courtade JA, Klimek-Abercrombie AM, Chen Y-C, Patel N, Lu PYT, Speake C, et al. Measurementof pro-islet amyloid polypeptide (1-48) in diabetes and islet transplants. J Clin Endocrinol Metab. 2017;102(7).

27. Kodama S, Davis M, Faustman DL. The therapeutic potential of tumor necrosis factor for autoimmune disease: a mechanistically based hypothesis. Cell Mol Life Sci. 2005 Aug;62(16):1850–62.

28. van Belle TL, Coppieters KT, von Herrath MG. Type 1 diabetes: etiology, immunology, and therapeutic strategies. Physiol Rev. 2011 Jan;91(1):79–118.

29. Flavell RA, Kratz A, Ruddle NH. The contribution of insulitis to diabetes development in tumor necrosis factor transgenic mice. Curr Top Microbiol Immunol. 1996;206:33–50.

30. Campbell IL, Cutri A, Wilkinson D, Boyd AW, Harrison LC. Intercellular adhesion molecule 1 is induced on isolated endocrine islet cells by cytokines but not by reovirus infection. Proc Natl Acad Sci U S A. 1989 Jun;86(11):4282–6.

31. Picarella DE, Kratz A, Li CB, Ruddle NH, Flavell RA. Transgenic tumor necrosis factor (TNF)-alpha production in pancreatic islets leads to insulitis, not diabetes. Distinct patterns of inflammation in TNF-alpha and TNF-beta transgenic mice. J Immunol. 1993 May;150(9):4136–50.

32. Kleijwegt FS, Laban S, Duinkerken G, Joosten AM, Zaldumbide A, Nikolic T, et al. Critical role for TNF in the induction of human antigen-specific regulatory T cells by tolerogenic dendritic cells. J Immunol. 2010 Aug;185(3):1412–8.

33. Padgett LE, Broniowska KA, Hansen PA, Corbett JA, Tse HM. The role of reactive oxygen species and proinflammatory cytokines in type 1 diabetes pathogenesis. Ann N Y Acad Sci. 2013 Apr;1281(1):16–35.

34. Koulmanda M, Bhasin M, Awdeh Z, Qipo A, Fan Z, Hanidziar D, et al. The Role of TNF-α in Mice with Type 1- and 2- Diabetes. von Herrath MG, editor. PLoS One. 2012 May;7(5):e33254.

35. Mastrandrea L, Yu J, Behrens T, Buchlis J, Albini C, Fourtner S, et al. Etanercept Treatment in Children With New-Onset Type 1 Diabetes: Pilot randomized, placebo-controlled, double-blind study. Diabetes Care. 2009 Jul;32(7):1244–9.

36. Herold KC, Hagopian W, Auger JA, Poumian-Ruiz E, Taylor L, Donaldson D, et al. Anti-CD3 Monoclonal Antibody in New-Onset Type 1 Diabetes Mellitus. N Engl J Med. 2002 May;346(22):1692–8.

37. Herold KC, Gitelman SE, Ehlers MR, Gottlieb PA, Greenbaum CJ, Hagopian W, et al. Teplizumab (anti-CD3 mAb) treatment preserves C-peptide responses in patients with new-onset type 1 diabetes in a randomized controlled trial: metabolic and immunologic features at baseline identify a subgroup of responders. Diabetes. 2013 Nov;62(11):3766–74.

38. Herold KC, Gitelman SE, Willi SM, Gottlieb PA, Waldron-Lynch F, Devine L, et al. Teplizumab treatment may improve C-peptide responses in participants with type 1 diabetes after the new-onset period: a randomised controlled trial. Diabetologia. 2013 Feb;56(2):391–400.

39. Campbell JE, Drucker DJ. Pharmacology, Physiology, and Mechanisms of Incretin Hormone Action. Cell Metab. 2013 Jun;17(6):819–37.

40. Bregenholt S, Møldrup A, Blume N, Karlsen AE, Nissen Friedrichsen B, Tornhave D, et al. The long-acting glucagon-like peptide-1 analogue, liraglutide, inhibits beta-cell apoptosis in vitro. Biochem Biophys Res Commun. 2005 May;330(2):577–84.

41. Rolin B, Larsen MO, Gotfredsen CF, Deacon CF, Carr RD, Wilken M, et al. The long-acting GLP-1 derivative NN2211 ameliorates glycemia and increases β-cell mass in diabetic mice. Am J Physiol - Endocrinol Metab. 2002 Oct;283(4):E745–52.

42. Rydén AK, Perdue NR, Pagni PP, Gibson CB, Ratliff SS, Kirk RK, et al. Anti-IL-21 monoclonal antibody combined with liraglutide effectively reverses established hyperglycemia in mouse models of type 1 diabetes. J Autoimmun. 2017 Nov;84:65–74.

43. Ghofaili K Al, Fung M, Ao Z, Meloche M, Shapiro RJ, Warnock GL, et al. Effect of Exenatide on ?? Cell Function After Islet Transplantation in Type 1 Diabetes. Transplantation. 2007 Jan;83(1):24–8.

44. Rother KI, Spain LM, Wesley RA, Digon BJ, Baron A, Chen K, et al. Effects of Exenatide Alone and in Combination With Daclizumab on  -Cell Function in Long-Standing Type 1 Diabetes. Diabetes Care. 2009 Dec;32(12):2251–7.

45. Kielgast U, Krarup T, Holst JJ, Madsbad S. Four Weeks of Treatment With Liraglutide Reduces Insulin Dose Without Loss of Glycemic Control in Type 1 Diabetic Patients With and Without Residual  -Cell Function. Diabetes Care. 2011 Jul;34(7):1463–8.

46. Buse JB, Rosenstock J, Sesti G, Schmidt WE, Montanya E, Brett JH, et al. Liraglutide once a day versus exenatide twice a day for type 2 diabetes: a 26-week randomised, parallel-group, multinational, open-label trial (LEAD-6). Lancet (London, England). 2009 Jul;374(9683):39–47.

47. Vilsbøll T, Zdravkovic M, Le-Thi T, Krarup T, Schmitz O, Courrèges J-P, et al. Liraglutide, a long-acting human glucagon-like peptide-1 analog, given as monotherapy significantly improves glycemic control and lowers body weight without risk of hypoglycemia in patients with type 2 diabetes. Diabetes Care. 2007 Jun;30(6):1608–10.

48. Garber A, Henry R, Ratner R, Garcia-Hernandez PA, Rodriguez-Pattzi H, Olvera-Alvarez I, et al. Liraglutide versus glimepiride monotherapy for type 2 diabetes (LEAD-3 Mono): a randomised, 52-week, phase III, double-blind, parallel-treatment trial. Lancet (London, England). 2009 Feb;373(9662):473–81.

49. Mathieu C, Zinman B, Hemmingsson JU, Woo V, Colman P, Christiansen E, et al. Efficacy and Safety of Liraglutide Added to Insulin Treatment in Type 1 Diabetes: The ADJUNCT ONE Treat-To-Target Randomized Trial. Diabetes Care. American Diabetes Association; 2016 Oct;39(10):1702–10.

# Attachments

## Attachment 1. Common Terminology Criteria for Adverse Events (CTCAE) Version 5

## Attachment 2. Victoza® (liraglutide) Prescribing Information

## Attachment 3. SIMPONI® (golimumab) Prescribing Information
