# Supplementary material for: Testing a new platform to screen disease-modifying therapy in type 1 diabetes
Source: PLoS One. 2023 Dec 14;18(12):e0293268. doi: 10.1371/journal.pone.0293268 (PMC10721089; doi:10.1371/journal.pone.0293268)
Supplement: S2 Protocol — (DOCX) [file pone.0293268.s003.docx]

**Protocol**

**Targeting Beta cell Dysfunction with Verapamil in Longstanding T1D**

Short Title: Waking Beta Cells: Verapamil

Version 2.0

07 January 2021

Investigator

Carla Greenbaum, MD

Table of Contents

**Section Page**

[1 Abbreviations 6](#_Toc39212754)

[2 Protocol Synopsis 8](#_Toc39212755)

[3 Background and rationale 9](#_Toc39212756)

[4 Study Objectives 13](#_Toc39212757)

[4.1 Primary Objective 13](#_Toc39212758)

[4.2 Secondary Objectives 13](#_Toc39212759)

[4.3 Exploratory Objectives 13](#_Toc39212760)

[4.4 Safety Objective 14](#_Toc39212761)

[5 Study Design 14](#_Toc39212762)

[5.1 Overview 14](#_Toc39212763)

[5.2 Endpoints 14](#_Toc39212764)

[5.2.1 Primary Endpoint 14](#_Toc39212765)

[5.2.2 Secondary/Exploratory Endpoints 15](#_Toc39212766)

[5.3 Eligibility 15](#_Toc39212767)

[5.3.1 Inclusion criteria 15](#_Toc39212768)

[5.3.2 Exclusion criteria 15](#_Toc39212769)

[5.4 Study Duration 16](#_Toc39212770)

[5.5 Study sites 16](#_Toc39212771)

[6 Study Procedures 16](#_Toc39212772)

[6.1 Screening Visits (visit numbers -2,-1) 16](#_Toc39212773)

[6.2 Administration of Study Drug 17](#_Toc39212774)

[6.2.1 Formulation 17](#_Toc39212775)

[6.2.2 Preparation and Accountability 17](#_Toc39212776)

[6.2.3 Treatment Visit (visit number 0) 17](#_Toc39212777)

[6.2.4 Dosing Windows 17](#_Toc39212778)

[6.3 Subsequent study visits (visits 1, 2, 3 and 4) 18](#_Toc39212779)

[6.4 Follow-up study visit (visit 5) 18](#_Toc39212780)

[6.5 Visit windows 18](#_Toc39212781)

[6.6 Assessments 19](#_Toc39212782)

[6.6.1 General Assessments 19](#_Toc39212783)

[6.6.2 Clinical Assessments 19](#_Toc39212784)

[6.6.3 Metabolic Assessments 19](#_Toc39212785)

[6.6.4 MRI Procedures 19](#_Toc39212786)

[6.6.5 Mechanistic Assessments 20](#_Toc39212787)

[7 PARTICIPANT SAFETY 20](#_Toc39212788)

[7.1 Benefit of Participation 20](#_Toc39212789)

[7.2 Risks of Participation and Mitigation of Risks 20](#_Toc39212790)

[7.2.1 Study procedures: Blood draws and Mixed Meal Tolerance Testing (MMTT) 20](#_Toc39212791)

[7.2.2 MRI procedure 20](#_Toc39212792)

[7.2.3 ECG Procedure 21](#_Toc39212793)

[7.2.4 Study medication: verapamil SR 21](#_Toc39212794)

[8 Adverse Event Reporting and Documentation 21](#_Toc39212795)

[8.1 Overview 21](#_Toc39212796)

[8.2 Definitions 22](#_Toc39212797)

[8.2.1 Adverse Event (AE) 22](#_Toc39212798)

[8.2.2 Adverse Reaction 22](#_Toc39212799)

[8.2.3 Serious Adverse Event (SAE) 22](#_Toc39212800)

[8.2.4 Unexpected or Unlisted Adverse Event 23](#_Toc39212801)

[8.3 Safety Assessments and Collection of Safety Data 23](#_Toc39212802)

[8.3.1 SAEs and Special Reporting Situations 24](#_Toc39212803)

[8.3.2 Reporting pregnancy 25](#_Toc39212804)

[9 Statistical Analysis Plan 25](#_Toc39212805)

[9.1 Endpoints: 25](#_Toc39212806)

[9.1.1 Primary Endpoint 25](#_Toc39212807)

[9.1.2 Secondary and Exploratory Endpoints 25](#_Toc39212808)

[9.2 Sample size: 26](#_Toc39212809)

[10 ETHICAL CONSIDERATIONS AND COMPLIANCE WITH GOOD CLINICAL PRACTICE 26](#_Toc39212810)

[10.1 Statement of Compliance 26](#_Toc39212811)

[10.2 Informed Consent 26](#_Toc39212812)

[10.3 Withdrawal of Subjects from the Study 27](#_Toc39212813)

[10.4 Privacy and Confidentiality 27](#_Toc39212814)

[11 Data Collection, Monitoring, and Sample Retention 27](#_Toc39212815)

[11.1 Data Collection Instruments 27](#_Toc39212816)

[11.2 Archival of Data 28](#_Toc39212817)

[11.3 Monitoring 28](#_Toc39212818)

[11.4 Sample Retention 28](#_Toc39212819)

[11.5 MRI Data Sharing 28](#_Toc39212820)

[12 Schedule of assessments 29](#_Toc39212821)

[13 References 30](#_Toc39212822)

[14 Attachments 32](#_Toc39212823)

[Attachment 1. Common Terminology Criteria for Adverse Events (CTCAE) Version 5 32](#_Toc39212824)

[Attachment 2. Verapamil SR Prescribing Information 32](#_Toc39212825)

# Abbreviations

**AE** Adverse event

**ALT** Alanine aminotransferase

**AST** Aspartate aminotransferase

**AUC** Area under the curve

**AV** Anterior Ventral

**BPM** Beats per minute

**BRI** Benaroya Research Institute at Virginia Mason

**BUN** Blood urea nitrogen

**CBC** Complete blood count

**CFR** Code of Federal Regulations

**CHF** Congestive heart failure

**CRF** Case report form

**CTCAE**  Common Terminology Criteria for Adverse Events

**DBP** Diastolic blood pressure

**DHHS** Department of Health and Human Services

**DKA** Diabetic ketoacidosis

**ECG** Electrocardiogram

**FDA** US Food and Drug Administration

**GCP** Good clinical practice

**GFR** Glomerular filtration rate

**HbA1c**  Glycosylated hemoglobin

**HR** Heart rate

**IAPP** Islet amyloid polypeptide

**ICH**  International Conference on Harmonization

**IDS** Investigational Drug Services

**IRB** Institutional review board

**LFT** Liver function test

**MMTT** Mixed-meal tolerance test

**MRI** Magnetic Resonance Imaging

**PI** Proinsulin

**PI/C** Proinsulin/C-peptide

**PK** Pharmacokinetic

**pro-IAPP** Prohormone IAPP_1-48_

**SAE** Serious adverse event

**SBP** Systolic blood pressure

**T1D**  Type 1 diabetes

**TXNIP** Thioredoxin-interacting protein

**ULN**  Upper limit of normal range

**VMMC**  Virginia Mason Medical Center

**WBC** White blood cell

# Protocol Synopsis

| Title | **Targeting Beta cell Dysfunction in Longstanding T1D** |
| --- | --- |
| Short Title | Waking Beta Cells |
| Sponsor Investigator | Carla Greenbaum, MD |
| Sponsor | Benaroya Research Institute at Virginia Mason |
| Conducted at | Benaroya Research Institute at Virginia Mason  Clinical Research Center  1201 Ninth Ave  Seattle,  WA 98101 |
| Accrual Objective | 15 subjects |
| Study Treatment | Three months of therapy with verapamil |
| Study Design | This application describes an open label, proof of concept study to determine whether verapamil can transiently improve beta cell function in those who do or do not secrete proinsulin and little/no C-peptide. |
| Primary Objective | To determine whether verapamil can transiently improve beta cell function in those individuals who do or do not secrete proinsulin and with C-peptide <0.017 pmol/mL, as assessed by increased C-peptide (primary outcome) or IAPP production, or a reduction in the PI/C or pro-IAPP/IAPP ratios |
| Primary Endpoints | Proportion of individuals with peak MMTT stimulated C-peptide >0.017 pmol/mL at 12 weeks. |
| Secondary Endpoints | 1. Changes in proinsulin, proinsulin to C-peptide ratio, IAPP and pro-IAPP between screening and 12-week MMTT 2. The proportion of those with C-peptide >0.017 pmol/mL at 12 weeks who are above and below that threshold after cessation of therapy 3. Relationship of metabolic measures to genetic, genomic and immunological response |
| Safety Endpoints | - Adverse events associated with verapamil treatment |
| Major Inclusion Criteria | - M/F 18-50 years of age, inclusive - ≥ 3 years from Diagnosis of T1D |

# Background and rationale

Type 1 diabetes mellitus (T1D) is an immune-mediated disease in which insulin-producing beta cells are completely or almost completely destroyed, resulting in life-long dependence on exogenous insulin. It is a chronic and potentially disabling disease that represents a major public health and clinical concern. The number of patients being diagnosed with T1D is increasing each year and is approaching an epidemic level in many countries (*1*).

For individuals living with T1D, continuous exogenous insulin therapy is needed to prevent ketoacidosis and other catabolic effects of insulin deficiency, to promote anabolism, and to maintain life. While there have been significant improvements in insulin analogs and insulin delivery systems, such as continuous subcutaneous insulin infusions with wearable pumps, continuous glucose monitoring, and hybrid closed loop systems, normal glucose control, particularly in children, is rarely achieved (*2*). Moreover, while the frequency of long-term complications is decreasing, individuals with T1D continue to have reduced life expectancy (*3-5*).

**Disease Modifying Therapy in T1D**

Since the first use of insulin in 1922, treatment of T1D has been targeted at controlling the symptoms and consequences of hyperglycemia. This is analogous to symptom control for individuals with rheumatoid and juvenile arthritis, where the aim of therapy was previously to control pain and/or provide devices to adapt to the presence of disability. Yet, control of symptoms of arthritis - and almost all other autoimmune disease - is no longer the primary aim of treatment; instead, the standard of care is disease-modifying therapy with the intent to fundamentally alter the course of the condition. This approach has dramatically changed the lives of those with these autoimmune diseases. The aim of disease-modifying therapy in T1D is the same: to move away from symptom management (e.g., glycemic control) as the mainstay of treatment and find approaches to alter the disease course – specifically to preserve beta cell function measured by C-peptide. Preservation of C-peptide post clinical diagnosis is associated with less short-term (hypoglycemia) and long-term (retinopathy, renal disease) complications. Islet transplant studies indicate that small amounts of endogenous secretion are insufficient for insulin independence but do prevent hypoglycemia.

Well-controlled clinical trials of disease modifying therapy in T1D have been largely limited to primary prevention (before development of autoantibodies), treatment at early, asymptomatic stages of disease (when antibodies are present with normal or abnormal glucose tolerance), or shortly after the onset of symptoms. At this time, four therapies with reasonable safety profiles have phase 2 clinical trial results demonstrating an altered disease course measured by a reduced fall in C-peptide during a mixed meal tolerance test (MMTT) in individuals shortly after onset of clinical disease (*6-9*). Each of these are immune therapies. There are few trials using therapies aimed at the beta cell itself to alter disease course at any stage of disease; yet there is increasing interest in testing such agents.


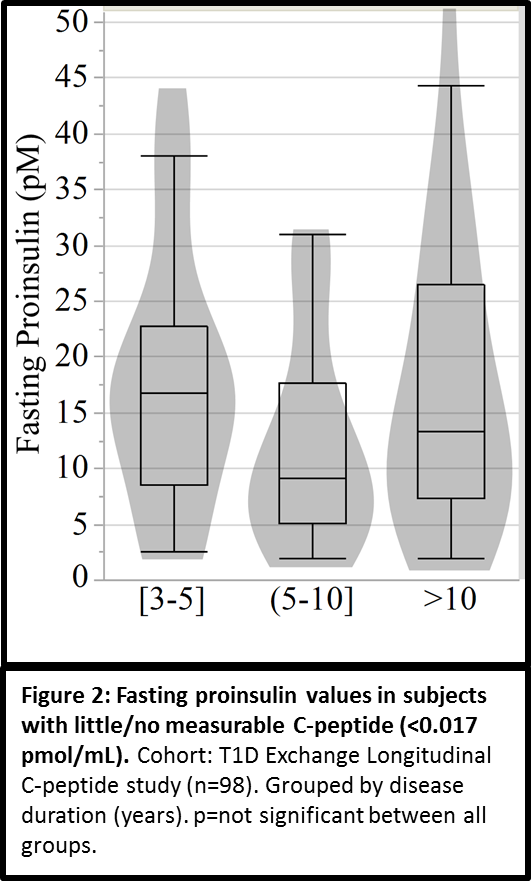
An alternative is to consider enrolling individuals further from diagnosis as there is a large population of adult individuals with longstanding T1D. This group has not previously been enrolled in trials of disease- modifying therapy because, until recently, there has been insufficient longitudinal information about the natural history of insulin secretion years from diagnosis to plan a trial; there has also been a lack of suitable alternative outcomes. We have just completed a longitudinal study of hundreds of individuals with longstanding type 1 diabetes examining the rate of fall of C-peptide assessed by regular MMTT over a 4-year period (manuscript in preparation). Not unexpectedly, among those with C-peptide at the start of the study, the rate of fall in this population is very low; thus, a very large number of participants would have to be studied over many years to detect an effect of therapy on preservation of C-peptide. Thus, one cannot conduct a small or short-term study of disease modifying therapy in those with longstanding T1D using preservation of C-peptide as an outcome.

Excitingly, however, our observation that almost all of the individuals three or more years from diagnosis with below detection limit C-peptide on MMTT secrete significant amounts of proinsulin suggests an alternative outcome measure that could allow for assessing the impact of therapies on beta cell health even in small studies. Since there are hundreds of thousands of individuals with longstanding T1D, if successful, this approach may allow for more rapid advancement of disease-modifying therapies.

We found that of 98 subjects with longstanding T1D and MMTT C-peptide <0.017 pmol/mL, 83 (85%) were secreting proinsulin above the limit of quantitation (2 pM). Proinsulin secretion was not associated with disease duration in this population (*Figure 2*). These data, which parallel recent findings in pancreata from T1D subjects (*10*), indicate the presence of beta cells (either residual, de-differentiated, or proliferating) in longstanding disease, but that they are not functioning properly. The data raise the question as to whether therapy can improve beta cell dysfunction in this population of subjects who have what we characterize as “sleeping” beta cells. Importantly, testing therapies in this population of individuals who have beta cells that are clearly present but severely dysfunctional or de-differentiated allows for a more direct assessment as to whether such cells are capable of secreting C-peptide. Evidence that this approach is of interest is provided in a study of longstanding T1D subjects awaiting islet transplant (median disease duration 27 years), many of whom showed increased C-peptide levels upon rapamycin induction therapy (*11*).

To better understand the health of the beta cell, we and others have been measuring proinsulin with the rationale that an increased proinsulin/insulin (or C-peptide) ratio implies defective prohormone processing and could be reflective of beta cell stress (*12*). The insulin processing pathway involves prohormone convertases 2 and 1/3 (*13, 14*). Impaired processing of proinsulin has been reported in type 2 diabetes (*15*), islet transplantation (*16*), and new-onset T1D (*17*). Studies from Belgium (*18*), Finland (*19*) and TrialNet (*20*) report impaired processing in antibody positive individuals prior to clinical T1D. Importantly, the proinsulin/C-peptide ratio has also been successfully used as an outcome measure in clinical interventions aimed to improve beta cell health. With respect to immunotherapy, in new-onset placebo-treated T1D patients, the fasting proinsulin/C-peptide ratio increased over time while this did not occur in those treated with cyclosporine (*21*), and anakinra (IL-1RA) treatment in type 2 patients significantly decreased the proinsulin/insulin ratio (*22*). A decrease in proinsulin/C-peptide ratio was also seen after gastric bypass surgery in obese adolescents coincident with an improved disposition index (beta cell function in relation to insulin sensitivity) (*23*). These studies support the notion that measurement of proinsulin/C-peptide in response to therapy will be indicative of improved beta cell function.

Other prohormones can also reflect beta cell stress and presence; like insulin/proinsulin, a pro-form of amylin (also known as islet amyloid polypeptide or IAPP) is also detectable in blood and associated with dysfunctional protein processing in the beta cell. Processing of prohormone IAPP_1-48_ (pro-IAPP) to IAPP involves the same enzymes as proinsulin processing - prohormone convertases 2 and 1/3 (*24, 25*). In longstanding T1D subjects from our adult cohort and a Canadian pediatric cohort, the pro-IAPP/IAPP ratio is increased compared to healthy subjects (*26*). Importantly, pro-IAPP was detectable in 100% of T1D subjects tested. In addition, like proinsulin, it is detectable in subjects without detectable fully processed hormone: 14/33 longstanding T1D subjects tested had detectable pro-IAPP in the absence of detectable IAPP (*26*). This suggests that inducing/increasing IAPP, or reducing the pro-IAPP/IAPP ratio, like proinsulin/C-peptide, may also reflect improvement of beta cell function in subjects with longstanding disease.

Pancreatic volume as measured by MRI may be an alternative measure of TID disease state and progression. Recent studies demonstrate reduced pancreatic volume in patients newly diagnosed with T1D and in autoantibody positive individuals prior to diagnosis (*27-30*), although with significant intersubject variability. These findings are interesting given that the beta cells constitute just 1-2% of total pancreatic volume. Particularly relevant to the proposed study, changes in pancreatic volume or altered MRI measured diffusion (*30*) might indicate disease state or predict response to therapy. In the proposed study, pancreatic MRIs will be conducted before and after 12 weeks of verapamil treatment to assess change in pancreatic volume and to assess the intersubject variability of pancreas size.

**Intervention:**

There are many therapies that have been proposed to improve beta cell function, by acting directly on the beta cell or by helping to improve the immune or metabolic milieu in the pancreas. Having identified a population of individuals with longstanding T1D who have severely dysfunctional or possibly de-differentiated, but not dead beta cells, one potentially can use this population in pilot studies to evaluate the impact of a series of therapies on beta cell function. In this protocol, we propose to test a therapy to support the beta cell, verapamil. This agent was selected due to pre-clinical and/or clinical data suggesting an impact on beta cell function in those who have beta cells capable of C-peptide secretion.

Thioredoxin-interacting protein (TXNIP) has been identified as a regulator of glucose and lipid metabolism. The TXNIP gene was identified as being upregulated in β cells, *in vitro*, in response to glucose stimulation and has been shown to be upregulated in diabetes (*31-33*). Moreover, overexpression of the *TXNIP* gene has been associated with increased β cells apoptosis (*33, 34*) and downregulation of this gene has been shown to improve insulin secretion and production (*35, 36*). A therapeutic agent that would downregulate TXNIP in the pancreatic β cells would have the potential to increase β cell function and potentially prevent further β cell loss.

Verapamil, an L-type calcium channel blocker, is an FDA approved anti-hypertensive drug that was identified in *in vitro* studies to decrease *TXNIP* gene expression in both mouse and human β cell (*37*). In mouse studies oral administration of verapamil showed reduced β cell death and improved β cell function (*37*). Additionally, a recent phase 2 trial conducted in recent onset T1D participants demonstrated that subjects administered daily oral verapamil had slower C-peptide decline over 12 months (*38*)(NCT02372253). Interestingly, in the first 3 months of verapamil use there was an increase in C-peptide, as measured during a MMTT (*38*). Here, because of this promising clinical and preclinical data, we propose to determine whether verapamil can improve beta cell function for those in long-standing T1D with little/no insulin secretion.

# Study Objectives

## Primary Objective

To determine whether verapamil can transiently improve beta cell function in individuals with severely dysfunctional or de-differentiated beta cells (MMTT C-peptide <0.017 pmol/mL and with detectable or undetectable proinsulin). This outcome will be assessed by increased C-peptide (primary outcome) or IAPP production, or a reduction in the PI/C or pro-IAPP/IAPP ratios.

## Secondary Objectives

1. To determine the relationship between impaired proinsulin processing with impaired processing of pro-IAPP in individuals with longstanding T1D.
2. To determine if the effect on beta cell function is sustained for 2 weeks after cessation of therapy.
3. To explore the relationship of metabolic measures to immunologic response to therapy.

## Exploratory Objectives

1. To measure pancreatic size in subjects with longstanding T1D, that are not producing any c-peptide.
2. To determine if there is a change in pancreatic volume as measured by MRI with 12 weeks of verapamil treatment.
3. To determine if there is a relationship of a change in pancreatic volume to response to therapy and to immune and metabolic markers.

## Safety Objective

Evaluate adverse effects associated with short-term administration of verapamil in T1D.

# Study Design

## Overview

This open label, proof of concept study will ask whether verapamil can transiently improve beta cell function in those who do or do not secrete proinsulin and little/no C-peptide. This is a mechanistic study aiming to determine whether severely dysfunctional or de-differentiated beta cells can be induced to secrete C-peptide; it is not designed to evaluate the clinical impact of these interventions.


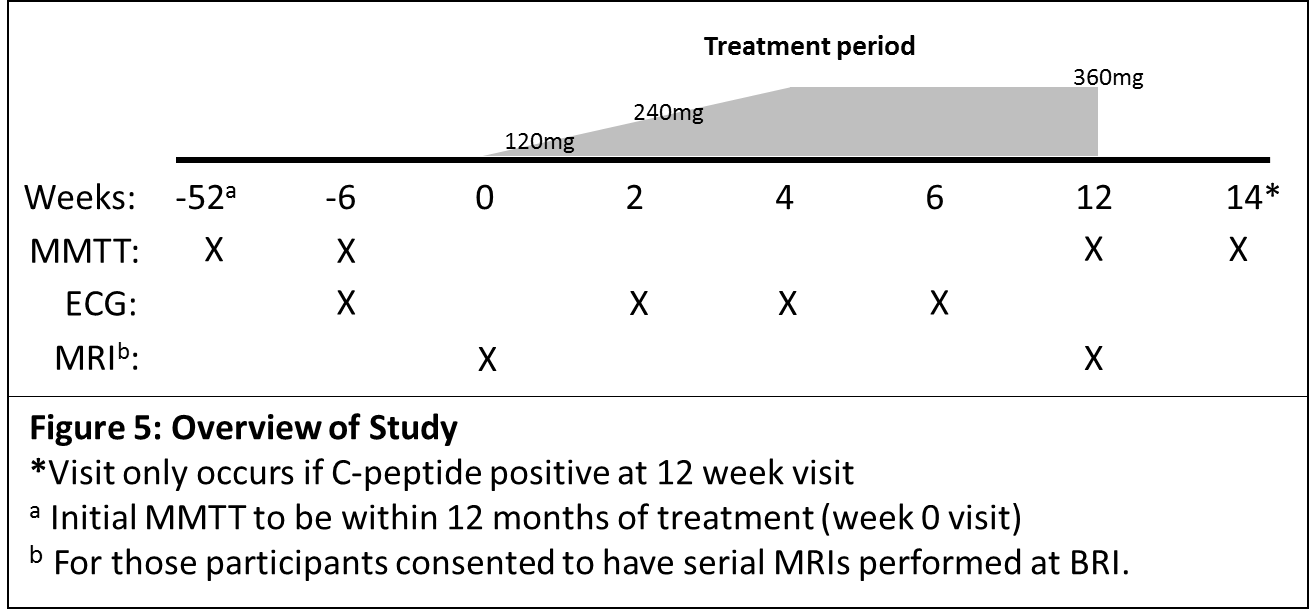
Subjects diagnosed with T1D who do or do not secrete proinsulin with little/no C-peptide and meeting other eligibility criteria will receive verapamil daily for 12 weeks as shown in Figure 5. Measures of beta cell function will be done before and after treatment, and if C-peptide is present at the end of therapy, follow-up evaluation will be done to assess duration of response.

## Endpoints

### Primary Endpoint

Proportion of individuals with peak MMTT stimulated C-peptide >0.017 pmol/mL at 12 weeks.

### Secondary/Exploratory Endpoints

1. Changes in proinsulin, proinsulin to C-peptide ratio, IAPP and pro-IAPP between screening and 12-week MMTT.
   - The proinsulin assays will be performed in a batch at the end of study.
2. The proportion of those with C-peptide > 0.017 pmol/mL at 12 weeks who are above and below that threshold after cessation of therapy at 14 weeks.
3. Exploratory:
   - Relationship of metabolic measures to genetic, genomic, and immunological response.
   - Pancreatic size in subjects with longstanding T1D.
   - Change in pancreatic volume and relationship of change in pancreatic volume to response to therapy and to immune and metabolic markers, as measured by MRI with 12 weeks of verapamil treatment.

## Eligibility

### Inclusion criteria

1. ≥ 3 years from Type 1 diabetes diagnosis or previously documented undetectable C-peptide
2. Males and females 18-50 years of age, inclusive
3. Peak MMTT stimulated C-peptide < 0.017 pmol/mL
4. Females of child-bearing potential must be willing to use effective birth control for 12 weeks
5. Willing and able to give informed consent for participation
6. HbA1c ≤ 8.5%

### Exclusion criteria

1. Concurrent use of non-insulin therapies aimed to control hyperglycemia or use within the past 30 days of initial qualifying MMTT (V-2).
2. Diagnosis of liver disease or elevated hepatic enzymes, as defined by ALT or AST> 1.5 x the upper limit of age-determined normal (ULN).
3. Renal disease, as defined by creatinine ≥1.5 mg/dL.
4. Hypersensitivity to verapamil or any component of the formulation.
5. Previous use of verapamil.
6. Known left ventricular dysfunction; bradycardia (HR <50 BPM) hypotension (systolic pressure <90 mm Hg); PR interval prolongation on EKG or any bradyarrhythmia (e.g. sick sinus syndrome, Anterior Ventral (AV) block); atrial flutter or fibrillation, and an accessory bypass tract (Wolff-Parkinson- White (WPW) syndrome, Lown-Ganong-Levine syndrome)
7. Uncompensated heart failure, fluid overload, myocardial infarction or evidence of ischemic heart disease or other serious cardiac disease as described in New York Heart Association (NYHA) Class III or IV criteria within the 12 weeks before randomization.
8. Use of beta blockers or medium-high dose statins: any dose of atorvastatin (Lipitor) or rosuvastatin (Crestor); simvastatin > 10 mg daily; lovastatin > 20 mg; pravastatin > 20 mg
9. Use of other medications which may increase the concurrent risk of verapamil use, including medications which utilize the cytochrome p450 enzyme pathway.
10. Females who are pregnant or lactating.
11. Receipt of an immune modulating biologic or investigational drug within 3 months or 5 half-lives before enrollment.
12. History of other clinically significant autoimmune disease except for celiac and stable thyroid disease.
13. Current use of any medication known to significantly influence glucose tolerance (e.g. oral steroids, atypical antipsychotics, diphenylhydantoin, niacin).
14. Any medical or psychological condition that in the opinion of the principal investigator would interfere with the safe completion of the trial. Conditions to consider include history of chronic GERD, chronic constipation, and chronic nausea.
15. Specific to MRI subjects: non-removable ferromagnetic materials or MRI not technically feasible (claustrophobia, movement disorder, obesity).

## Study Duration

Enrollment is anticipated to occur within 18 months. The rate of enrollment may vary as data is collected and evaluated.

## Study sites

The study will be conducted at the Benaroya Research Institute (BRI) at Virginia Mason in Seattle, WA.

# Study Procedures

## Screening Visits (visit numbers -2,-1)

Potentially eligible participants will undergo screening visit(s) to determine eligibility for the study.   Eligible individuals must have two consecutive MMTTs within a 12-month window from the treatment visit (V0) with peak C-peptide <0.017 pmol/mL. One of these can be conducted under a different study protocol. Review of systems with directed exam, including vital signs, medical history, and pregnancy test (as appropriate) will be done. ECG and clinical labs will be performed. Any participant whose other laboratory values exclude the participant from the study may undergo repeat assessments.  In these cases, two subsequent tests are required to confirm eligibility.

## Administration of Study Drug

### Formulation

Verapamil sustained release (SR) is a calcium ion influx inhibitor (slow channel blocker or calcium ion antagonist). It is FDA approved for treatment of hypertension, angina, supraventricular tachycardia and headache prophylaxis. Dosing range is 120mg-480mg daily, with a maximum dose of 480mg daily. For this trial, the drug will be administered initially as 120 mg orally daily with increase to 240 mg daily at approximately 14 days and then to 360 mg daily 14 days later as tolerated. Participants will have the option of maintaining a dose of 240mg daily if side effects prevent dose escalation to 360 mg daily.

### Preparation and Accountability

Verapamil will be stored at the VMMC Investigational Pharmacy. Records of the disposition of the investigational product, including the date and quantity of drug that was received, the participants to whom drug was dispensed (by participant accounting), and an account of any drug accidentally or deliberately destroyed will be kept.

A drug administration log will be kept current for each participant and will contain the identification of each participant and the date and quantity of drug dispensed. All remaining unused investigational product will be returned to the Investigational Drug Services (IDS) at Virginia Mason and destroyed according to the standard operating procedures of the IDS.

### Treatment Visit (visit number 0)

Criteria for dosing: Review of systems with directed exam, including vital signs, medical history, and pregnancy test (as appropriate) will be done at the dosing visit. Investigator will confirm that participant still meets all eligibility criteria prior to dosing.

Verapamil SR be initiated at a dose of 120 mg at the initial treatment visit. Individuals will be instructed on self-administration of study drug. They will be dispensed study drug for home administration. Participants who consent to MRI will have a baseline MRI within 2 weeks before or after this visit.

### Dosing Windows

All attempts should be made to dose on the scheduled target dosing day, however due to scheduling and other events this may not be possible.

Verapamil SR is dosed once per day. Missed treatment doses will not made up and participants will take their next dose according to the dosing schedule. If Visit 4 is unable to be conducted within the preferred window, additional study drug will be provided for up to an additional 5 days to assure individuals are on study drug at the time of the visit.

## Subsequent study visits (visits 1, 2, 3 and 4)

Review of systems and directed exam including AE assessments will be done. Clinical and mechanistic samples will be obtained. Participants will undergo an ECG at visits 1, 2 and 3. Visit 4 is the primary outcome visit. Individuals will undergo an MMTT at visit 4. Participants who consent to MRI may undergo an MRI within 2 weeks before or after visit 4.

## Follow-up study visit (visit 5)

Individuals who have C-peptide >0.017 pmol/mL at 12 weeks will return for follow-up assessment off study drug at 14 weeks. Review of systems and directed exam including AE assessments will be done. Clinical and mechanistic samples will be obtained. Individuals will undergo an MMTT.

## Visit windows

**First qualifying MMTT (V-2):** The first qualifying MMTT must occur within 12 months of the treatment visit (V0). It may occur within or outside the study.

**Screening Visit (V-1):** The screening visit and second qualifying MMTT must occur within 42 days of the treatment visit (V0).

**Treatment visit (V0):** The initial treatment must begin within 42 days of the screening visit (V-1) MMTT.

**Study Visits (V1, V2, V3 and V4):** within 3 days on either side of the target date for visits 1, 2 and 3 and within 5 days on either side of target date for visit 4. The aim is to have participants on study drug at the time of Visit 4 (Week 12).

- The last dose of study drug will be at Visit 4. If visit 4 is unable to occur within the preferred window due to scheduling or other events such as intercurrent illness, up to 2 weeks of additional study drug may be provided.

**Follow up visit (V5):** If a participant is C-peptide positive at study visit 4 then they will have a follow up visit at 14 weeks that must occur within 7 days on either side of the target date.

Section 12 presents the schedule of events for this trial.

## Assessments

### General Assessments

- Informed consent: written informed consent will be obtained from the participant before any study assessments or procedures are performed
- Eligibility criteria: eligibility for study participation will be assessed during the screening period
- Medical History: Relevant medical history, including history of current and previous disease, and review of systems will be obtained. Adverse events will be assessed. Vital signs including blood pressure and heart rate will be collected. A directed physical exam will be conducted as indicated by medical history
- Concomitant medications: concomitant medications and their indications will be recorded

### Clinical Assessments

- Islet autoantibodies: Antibody levels will be measured for all subjects at screening unless evidence of one or more islet antibodies has been previously documented.
- Basic Metabolic Panel: Electrolytes (sodium, potassium, chloride, total CO2, calcium), glucose, blood urea nitrogen (BUN), creatinine, GFR.
- Liver tests (AST and ALT)
- Hematology: Includes RBC, hematocrit, hemoglobin, platelet count, WBC and differential,
- Pregnancy testing: Females with reproductive capacity will undergo a urine pregnancy test at screening and subsequent study visits
- ECG

### Metabolic Assessments

- 2-hour mixed meal tolerance test with measurements of C-peptide, proinsulin, pro-IAPP and IAPP, and glucose
- HbA1c

### 6.6.4 MRI Procedures

During the MRI scanning session, participants will do the following:

- Provide MRI-relevant medical history to the MRI technician per Virginia Mason Hospital radiology department protocol
- Receive information on how to alert the staff if in need of assistance
- Lie still in supine position for approximately 40 minutes
- Hold breath for up to 25 seconds
- Wear earplugs and/or headphones if desired

### 6.6.5 Mechanistic Assessments

**Mechanistic samples:** Samples will be drawn at each study visit which will become part of the Benaroya Research Institute’s Immune Mediated Disease Repository. As such, they will be used for genetic, genomic, and immunologic studies related to T1D and the mechanisms of action of the therapies being tested.

# PARTICIPANT SAFETY

## Benefit of Participation

This study will not provide any direct benefit to the study subjects. Future patients may benefit from this study by virtue of knowledge gained about ability of severely dysfunctional beta cells to recover function.

## Risks of Participation and Mitigation of Risks

### Study procedures: Blood draws and Mixed Meal Tolerance Testing (MMTT)

There is minimal risk as a result of study procedures such as blood draw and IV placement which may result in discomfort or bruising and rarely phlebitis.

### MRI procedure

There is minimal risk to an MRI. Risks include claustrophobia or discomfort while positioned in the scanner and the risk of incidental findings leading to anxiety and financial costs. No MRI contrast agents or nuclides will be administered for any of the MRI scans. Prior to MRI, subjects will be screened for the presence of ferromagnetic materials per standard VM protocol. All MRI instruments are FDA approved for clinical use.

After entering the MRI room, participants will be given earplugs and/or headphones. They will be instructed to lie supine. Participants may be asked to hold their breath for up to 25 seconds. Standard communication and instruction are performed via a built-in intercom system. Subjects will be encouraged to terminate the session if they become uncomfortable. A typical MRI experimental session takes approximately 40 minutes. In the event of an injury or emergency, immediate and necessary care for the injury will be completed by onsite emergency personnel.

Plan for incidental MRI findings: Participants will be informed of the risk of incidental findings during the consent discussion. For the purposes of the study, pancreatic volume will be evaluated by study personnel, but there will be no clinical MRI assessment. As such, MRIs will have a “safety read” performed by a Virginia Mason radiologist to ensure that any clinically important finding will be discussed with participants and appropriate follow up will be coordinated by study providers.

### ECG Procedure

There is no risk to the ECG procedure. Plan for incidental ECG findings: Participants will be informed of the risk of incidental findings during the consent discussion. Any clinically important findings will be discussed with participants and appropriate follow up will be coordinated by study providers.

### Study medication: verapamil SR

Common side effects:

The following reactions to orally administered verapamil occurred at rates greater than 1.0% or occurred at lower rates but appeared clearly drug-related in clinical trials:

Constipation (7.3%), Dyspnea (1.4%), Dizziness (3.3%), Bradycardia (HR < 50/min) (1.4%), Nausea (2.7%), Hypotension (2.5%), AV Block (total 1°, 2°, 3°) 1.2%; (2° and 3°) (0.8%), Headache (2.2%), Edema (1.9%), CHF, Pulmonary Edema (1.8%), Rash (1.2%), Fatigue (1.7%), Flushing (0.6%).

Specific concerns and risk mitigation for verapamil SR:

Most side effects are dose related and should reduce with gradual titration and continued use.

- Cardiac and cardiac related side effects (dizziness, bradycardia, hypotension, AV block, CHF) will be reduced by exclusion of at-risk participants and by regular monitoring of symptoms, vital signs, and ECG. The maximum dose may be 240 mg daily if side effects prevent dose escalation.
- GI side effects (constipation and nausea) will be mitigated by gradual dose escalation and with a maximum dose of 240 mg daily if side effects prevent dose escalation. Participants with a history of chronic constipation or chronic nausea may be excluded at screening per investigator discretion.
- Hypoglycemia: Concomitant use of insulin with verapamil may result in hypoglycemia. Insulin dose adjustments may be recommended by a qualified staff member during regular phone or text check-ins and/or at study visits

# Adverse Event Reporting and Documentation

## Overview

As the sponsor of the Study, the Sponsor Investigator shall be solely responsible for complying, within the required timelines, with any safety reporting obligation to competent Health Authorities, IRB/ECs and any participating co- or sub-investigators, as defined in applicable laws and regulations.

Safety will be monitored by the Clinical Investigator(s). Participant concerns and symptoms will be formally assessed at each visit and the study team in contact in between visits as clinically indicated.

## Definitions

### Adverse Event (AE)

An adverse event is any untoward medical occurrence in a clinical study in which a subject is administered a medicinal (investigational or non-investigational) product. An adverse event does not necessarily have a causal relationship with the treatment. An adverse event can therefore be any unfavorable and unintended sign (including an abnormal finding), symptom, or disease temporally associated with the use of a medicinal (investigational or non- investigational) product, whether or not related to that medicinal (investigational or non-investigational) product. (Definition per International Conference on Harmonisation [ICH]). This includes any occurrence that is new in onset or aggravated in severity or frequency from the baseline condition, or abnormal results of diagnostic procedures, including laboratory test abnormalities.

### Adverse Reaction

An adverse reaction means any adverse event caused by a drug. Adverse reactions are a subset of all suspected adverse reactions for which there is reason to conclude that the drug caused the event. Suspected adverse reaction means any adverse event for which there is a reasonable possibility that the drug caused the adverse event. For the purposes of safety reporting, “reasonable possibility” means there is evidence to suggest a causal relationship between the drug and the adverse event. A suspected adverse reaction implies less certainty about causality than an adverse reaction, which means any adverse event caused by a drug. Examples of evidence that suggest a causal relationship (reasonable possibility) between the drug and the adverse event include:

- A single occurrence of an event that is uncommon and known to be strongly associated with drug exposure.
- One or more occurrences of an event that is not commonly associated with drug exposure but is otherwise uncommon in the populations exposed to the drug.

### Serious Adverse Event (SAE)

A serious adverse event (SAE) or reaction is defined as “any adverse event occurring at any dose that suggests a significant hazard, contraindication, side effect, or precaution.” An adverse event or suspected adverse reaction is considered “serious” if, in the view of the sponsor investigator, it results in any of the following outcomes:

- Death. A death that occurs during the study or that comes to the attention of the investigator during the protocol-defined follow-up after the completion of therapy must be reported whether it is considered treatment related or not.
- A life-threatening adverse event. A life-threatening event is any adverse therapy experience that, in the view of the investigator, places the participant at immediate risk of death from the reaction as it occurred.
- Inpatient hospitalization or prolongation of existing hospitalization with the exception of hospitalization relating to glycemic control in type 1 diabetes.
- Persistent or significant incapacity or substantial disruption of the ability to conduct normal life functions.
- Congenital anomaly or birth defect.
- Important medical events* that may not result in death, be life-threatening, or require hospitalization may be considered serious when, based upon appropriate medical judgment, they may jeopardize the patient or subject and may require medical or surgical intervention to prevent one of the outcomes listed above.
- Is a suspected transmission of any infectious agent via a medicinal product.

*Medical and scientific judgment should be exercised in deciding whether expedited reporting is also appropriate in other situations, such as important medical events that may not be immediately life threatening or result in death or hospitalization but may jeopardize the subject or may require intervention to prevent one of the other outcomes listed in the definition above. These should usually be considered serious.

### Unexpected or Unlisted Adverse Event

An adverse event/reaction is considered unexpected when the nature (specificity) or severity of the event is not consistent with the risks described in the applicable product information, Investigator’s Brochure or the informed consent document. Unexpected refers to an experience that has not been previously observed. This includes events that occur more frequently than expected.

## Safety Assessments and Collection of Safety Data

This is an exploratory study designed to assess the effects of 12 weeks of therapy on β cell function. All adverse events, regardless of causality, will be collected beginning with screening (V-1).

The Investigator(s) will treat participants with adverse events appropriately and observe them at suitable intervals until the events resolve or stabilize. Adverse events may be discovered through:

- observation of the participant.
- questioning the participant.
- unsolicited complaints by the participant.

All serious adverse events, adverse events of special interest, product quality complaints, and special situations including pregnancies, whether serious or non-serious, related or not related, following exposure to study drug will be documented by the Sponsor Investigator (or sub-Investigators) and recorded and maintained in subject’s source documents. Serious and Unexpected adverse events deemed possibly or probably drug related will be reported to the IRB per requirements within 24 hours of notification of the event. Events will be assessed and reported consistent with the ICH Guideline for Good Clinical Practice, 21 CFR 312.32 for expedited safety reporting, per the guidance of the DHHS Office for Human Research Protections (OHRP), and according to any reporting requirements outlined in this protocol.

Events with the exception of hypoglycemia or hyperglycemia will be graded per the National Cancer Institute’s Common Terminology Criteria for Adverse Events Version 5.0 (published November 27, 2017) They will be characterized as follows: duration (start and stop dates and times), severity/grade, outcome, treatment and relationship to study drug (not, unlikely, possibly, probably, or definitely related) will be recorded on source documents. The batch and lot number of the product will be recorded for all patients, including dose and date of administration. An adverse hypoglycemic event is defined as one resulting in loss of consciousness, seizure, or requiring assistance of others due to altered state of consciousness. An adverse hyperglycemic event is one resulting in diabetic ketoacidosis (DKA).

### SAEs and Special Reporting Situations

All serious adverse events that have not resolved by the end of the study, or that have not resolved upon discontinuation of the subject’s participation in the study, must be followed until any of the following occurs:

- The event resolves.
- The event stabilizes.
- The event returns to baseline if a baseline value/status is available.
- The event can be attributed to agents other than the study drug or to factors unrelated to study conduct; or

It becomes unlikely that any additional information can be obtained (subject or health care practitioner refusal to provide additional information, lost to follow-up after demonstration of due diligence with follow-up efforts).

### Reporting pregnancy

Any subject who becomes pregnant during the study must be promptly withdrawn from the study and discontinue further participation.

The investigator should be informed immediately of any pregnancy in the participant or a partner pregnancy of a male participant occurring during the treatment period. The investigator should be available to counsel the participant or refer the participant (or partner) for counseling to discuss possible risks to the pregnancy and fetus. Pregnancy, pregnancy complication or pregnancy termination would be recorded as an AE. The pregnancy outcome will be recorded.

# Statistical Analysis Plan

## Endpoints:

### Primary Endpoint

The primary endpoint is the proportion of individuals with peak MMTT stimulated C-peptide >0.017 pmol/mL at 12 weeks.

### Secondary and Exploratory Endpoints

Analysis may include data collected from other studies. Secondary endpoints include changes in proinsulin, proinsulin to C-peptide ratio, IAPP and pro-IAPP between screening and 12-week MMTT. The duration of effect will be determined by assessing the proportion of those with C-peptide >0.017 pmol/mL at 12 weeks who are above and below that threshold 2 weeks after cessation of therapy.

Exploratory endpoints will include the relationship between the metabolic effects of therapy and future genetic, genomic, and immunologic studies using stored samples; change in pancreatic volume with 12 weeks of verapamil treatment; relationship of change in pancreatic volume to response to therapy and to immune and metabolic markers. In each MRI scan, the pancreas will by outlined freehand on contiguous slices of the anatomical image. The resulting mask will be used to calculate pancreas volume and applied to parametric MRI maps to extract mean values for each measure.

## Sample size:

This trial is a pilot, mechanistic, proof of concept study to determine whether severely dysfunctional or de-differentiated beta cells can be induced to secrete C-peptide. As a proof of concept study, we wanted to detect such an outcome in even a small proportion of individuals. Thus, our study is powered to detect a positive outcome (defined as the presence of MMTT stimulated C-peptide >0.017 pmol/mL at 12 weeks) in at least 20% of participants for each study. Studying 15 participants gives 83% power to detect a positive outcome in 20% of participants, at an alpha of 0.05. This number of participants would also give 90% power to detect a larger proportion of individuals with a positive outcome (25-30% of subjects) at the same alpha.

Individuals unable to tolerate or who are non-compliant with study medication through 12 weeks or who do not undergo week 12 MMTT will be replaced. It is important to note that this is unlikely as the study medication is widely used clinically, yet if this occurs in 50% or more of the first 10 subjects in the study, the study may be paused or stopped and feasibility re-evaluated.

# ETHICAL CONSIDERATIONS AND COMPLIANCE WITH GOOD CLINICAL PRACTICE

## Statement of Compliance

This study will be conducted at Benaroya Research Institute at Virginia Mason in Seattle, WA. The study will be conducted in compliance with the protocol and consistent with current Good Clinical Practices (GCP), adopting the principles of the Declaration of Helsinki, and all applicable regulatory requirements (ICH E6, 45CFR46, and FDA 21CFR sections 11, 50, 56, 312).

Prior to study initiation, the protocol and the informed consent documents will be reviewed and approved by the Benaroya Research Institute at Virginia Mason Institutional Review Board (IRB). Any amendments to the protocol or consent materials will also be approved by the IRBs.

## Informed Consent

The process of assuring that individuals are making an informed decision about participating in this study includes both verbal and written communication. All participants must read, sign, and date a consent form before participating in the study, taking the study drug, and/or undergoing any study-specific procedures.

The informed consent form must be updated or revised whenever important new safety information is available, whenever the protocol is amended, and/or whenever any new information becomes available that may affect participation in the trial.

A copy of the informed consent will be given to a prospective participant for review.

## Withdrawal of Subjects from the Study

A subject may be withdrawn from the study at any time if the subject, the investigator, or the Sponsor feels that it is not in the subject’s best interest to continue.

Participants will be withdrawn and may be replaced from the study if they develop hypotension (SBP < 90mmHg, DBP < 50mmHg), bradycardia (HR < 50 bpm), or EKG changes (PR- or QT- interval prolongation).

All subjects are free to withdraw from participation at any time, for any reason, specified or unspecified, and without prejudice.

Reasonable attempts will be made by the investigator to provide a reason for subject withdrawals.  The reason for the subject’s withdrawal from the study will be specified in the subject’s source documents.

Individuals unable to tolerate verapamil SR at least 240 mg daily by four weeks after the initial treatment visit, or those who are subsequently known to be non-compliant or unable to tolerate 3 months of study medication, will be withdrawn from study and may be replaced. Participants who miss 5 or more consecutive doses of medication will be withdrawn and may be replaced. All subjects who have received any study medication will be considered in the safety evaluation cohorts. Those with adverse events possibly related to study medication will be followed as clinically appropriate until resolution.

## Privacy and Confidentiality

A participant’s privacy and confidentiality will be respected throughout the study. Each participant will be assigned a sequential identification number. This number, rather than the participant’s name, will be used in data analysis and reports

# Data Collection, Monitoring, and Sample Retention

## Data Collection Instruments

The Clinical Investigators will prepare and maintain adequate and accurate source documents designed to record all observations and other pertinent data for each subject treated with the study drug. Data from source documents will be entered into protocol specific case report forms. The Investigator is responsible for all information collected on subjects enrolled in this study.

## Archival of Data

The database is safeguarded against unauthorized access by established security procedures; appropriate backup copies of the database and related software files will be maintained.  Databases are backed up by the database administrator in conjunction with any updates or changes to the database.

## Monitoring

This study will be monitored by representatives of the Benaroya Research Institute Clinical Research Program Administrative Office according to the U.S. CFR Title 21 Parts 50, 56, and 312 and ICH Guidelines for GCP (E6).

All study documents (patient files, signed informed consent forms, copies of CRFs, Study File Notebook, etc.) will be available for monitors and maintained for at least two years after the study is completed.

## Sample Retention

Specimens collected in this trial will be deposited into the BRI sample repository and may be used to evaluate additional responses as new research tools become available or exploratory hypotheses are generated. Residual specimens may be used by investigators at BRI or at collaborating institutions for development of new assays or for comparisons across studies.

## MRI Data Sharing

De-identified and coded MRI data from BRI-consented subjects will be shared with the University of Texas Austin for pancreas volume estimation. All de-identified imaging data will be stored on a secure server with restricted access.

# Schedule of assessments

| Visit number | -2^1^ | -1 | 0 | 1 | 2 | 3 | 4 | 5^2,3^ |
| --- | --- | --- | --- | --- | --- | --- | --- | --- |
| Time point (week) | Initial MMTT | Screening visit | Initial treatment visit | Week 2 | Week 4 | Week 6 | Week 12 Primary Outcome visit | Week 14 Follow-up |
| Time point (day) | Within 12 months of visit 0 | Within 42 days of visit 0 | 0 | 14±3 days | 28±3 days | 42±3 days | 84±5 days | 98±7 days |
| General assessments (initial and interim history and directed physical) | X | X | X | X | X | X | X | X |
| Adverse events assessment* |  | X | X | X | X | X | X | X |
| MMTT | X | X |  |  |  |  | X | X |
| Islet autoantibodies^6^ | X |  |  |  |  |  |  |  |
| HbA1c | X | X | X |  |  |  | X | X |
| Concomitant medications including insulin dose* | X | X | X | X | X | X | X | X |
| Urine Pregnancy Test^5^ | X | X | X | X | X | X | X | X |
| Basic Metabolic Panel |  | X |  |  |  |  |  |  |
| AST, ALT |  | X^4^ |  |  |  |  |  |  |
| CBC with differential  and platelet count |  | X^4^ | X | X | X | X | X | X |
| ECG |  | X |  | X | X | X |  |  |
| MRI^7^ |  |  | X |  |  |  | X |  |
| Samples for mechanistic studies | X | X | X | X | X | X | X | X |

*Every other week contacts between visit 3 and visit 4 to query for adverse events and compliance with study medication.

1. Initial MMTT may be done within 12 months of initial treatment visit
2. Visit 5 only if MMTT C-peptide at visit 4 >0.017 pmol/mL.
3. Any adverse events or abnormal clinical laboratory values possibly related to study medication or procedures will be followed off study medication up to visit 5 or as clinically appropriate.
4. Screening lab results may be obtained at Initial MMTT (visit -2) if scheduled within 42 days of Initial Treatment Visit.
5. Females with reproductive capacity only.
6. Islet autoantibodies testing to be performed only on participants that do not have documented results from previous testing.
7. For those participants that consent to MRI, MRI visit will occur within 2 weeks prior to study Visit 0 or within 2 weeks post Visit 4.

# References

1. E. J. Mayer-Davis *et al.*, Incidence Trends of Type 1 and Type 2 Diabetes among Youths, 2002-2012. *N Engl J Med* **376**, 1419-1429 (2017).

2. K. M. Miller *et al.*, Current State of Type 1 Diabetes Treatment in the U.S.: Updated Data From the T1D Exchange Clinic Registry. *Diabetes Care* **38**, 971-978 (2015).

3. V. Gagnum *et al.*, Low Incidence of End-Stage Renal Disease in Childhood-Onset Type 1 Diabetes Followed for Up to 42 Years. *Diabetes Care* **41**, 420-425 (2018).

4. M. Lind *et al.*, Glycemic control and excess mortality in type 1 diabetes. *N Engl J Med* **371**, 1972-1982 (2014).

5. M. K. Ali, K. M. Bullard, E. W. Gregg, Achievement of goals in U.S. Diabetes Care, 1999-2010. *N Engl J Med* **369**, 287-288 (2013).

6. K. C. Herold *et al.*, A single course of anti-CD3 monoclonal antibody hOKT3gamma1(Ala-Ala) results in improvement in C-peptide responses and clinical parameters for at least 2 years after onset of type 1 diabetes. *Diabetes* **54**, 1763-1769 (2005).

7. M. R. Rigby *et al.*, Targeting of memory T cells with alefacept in new-onset type 1 diabetes (T1DAL study): 12 month results of a randomised, double-blind, placebo-controlled phase 2 trial. *Lancet Diabetes Endocrinol* **1**, 284-294 (2013).

8. M. D. Pescovitz *et al.*, Rituximab, B-lymphocyte depletion, and preservation of beta-cell function. *N Engl J Med* **361**, 2143-2152 (2009).

9. T. Orban *et al.*, Co-stimulation modulation with abatacept in patients with recent-onset type 1 diabetes: a randomised, double-blind, placebo-controlled trial. *Lancet* **378**, 412-419 (2011).

10. T. Rodriguez-Calvo *et al.*, Increase in Pancreatic Proinsulin and Preservation of Beta Cell Mass in Autoantibody Positive Donors prior to Type 1 Diabetes Onset. *Diabetes*, (2017).

11. L. Piemonti *et al.*, Beta cell function during rapamycin monotherapy in long-term type 1 diabetes. *Diabetologia* **54**, 433-439 (2011).

12. D. L. Eizirik, M. Miani, A. K. Cardozo, Signalling danger: endoplasmic reticulum stress and the unfolded protein response in pancreatic islet inflammation. *Diabetologia* **56**, 234-241 (2013).

13. M. Furuta *et al.*, Incomplete processing of proinsulin to insulin accompanied by elevation of Des-31,32 proinsulin intermediates in islets of mice lacking active PC2. *J Biol Chem* **273**, 3431-3437 (1998).

14. X. Zhu *et al.*, Severe block in processing of proinsulin to insulin accompanied by elevation of des-64,65 proinsulin intermediates in islets of mice lacking prohormone convertase 1/3. *Proc Natl Acad Sci U S A* **99**, 10299-10304 (2002).

15. W. K. Ward *et al.*, Disproportionate elevation of immunoreactive proinsulin in type 2 (non-insulin-dependent) diabetes mellitus and in experimental insulin resistance. *Diabetologia* **30**, 698-702 (1987).

16. A. M. Klimek *et al.*, Impaired proinsulin processing is a characteristic of transplanted islets. *Am J Transplant* **9**, 2119-2125 (2009).

17. R. A. Watkins *et al.*, Proinsulin and heat shock protein 90 as biomarkers of beta-cell stress in the early period after onset of type 1 diabetes. *Transl Res* **168**, 96-106 e101 (2016).

18. I. Truyen *et al.*, Proinsulin levels and the proinsulin:c-peptide ratio complement autoantibody measurement for predicting type 1 diabetes. *Diabetologia* **48**, 2322-2329 (2005).

19. M. E. Roder *et al.*, Disproportionately elevated proinsulin levels precede the onset of insulin-dependent diabetes mellitus in siblings with low first phase insulin responses. The Childhood Diabetes in Finland Study Group. *J Clin Endocrinol Metab* **79**, 1570-1575 (1994).

20. E. K. Sims *et al.*, Elevations in the Fasting Serum Proinsulin-to-C-Peptide Ratio Precede the Onset of Type 1 Diabetes. *Diabetes Care* **39**, 1519-1526 (2016).

21. O. Snorgaard, S. G. Hartling, C. Binder, Proinsulin and C-peptide at onset and during 12 months cyclosporin treatment of type 1 (insulin-dependent) diabetes mellitus. *Diabetologia* **33**, 36-42 (1990).

22. C. M. Larsen *et al.*, Interleukin-1-receptor antagonist in type 2 diabetes mellitus. *N. Engl. J. Med* **356**, 1517-1526 (2007).

23. T. H. Inge *et al.*, Insulin Sensitivity and beta-Cell Function Improve after Gastric Bypass in Severely Obese Adolescents. *J Pediatr* **167**, 1042-1048 e1041 (2015).

24. J. Wang *et al.*, The prohormone convertase enzyme 2 (PC2) is essential for processing pro-islet amyloid polypeptide at the NH2-terminal cleavage site. *Diabetes* **50**, 534-539 (2001).

25. L. Marzban *et al.*, Role of beta-cell prohormone convertase (PC)1/3 in processing of pro-islet amyloid polypeptide. *Diabetes* **53**, 141-148 (2004).

26. J. A. Courtade *et al.*, Measurement of Pro-Islet Amyloid Polypeptide (1-48) in Diabetes and Islet Transplants. *J Clin Endocrinol Metab* **102**, 2595-2603 (2017).

27. G. A. Gaglia JL, Harisinghani M, et al., Noninvasive imaging of pancreatic islet inflammation in type 1A diabetes patients. *J Clin Invest* **121**, 442-445 (2011).

28. T. S. Williams AJ, Sequeiros IM, et al., Pancreatic volume is reduced in adult patients with recently diagnosed type 1 diabetes. *J Clin Endocrinol Metab* **97**, E2109-E2113 (2012).

29. W. C. Campbell-Thompson M, Montgomery EL, et al. , Pancreas organ weight in individuals with disease-associated autoantibodies at risk for type 1 diabetes. *JAMA* **308**, 2337-2339 (2012).

30. W. J. Virostko J, Hilmes M, et al., Pancreas Volume Declines During the First Year After Diagnosis of Type 1 Diabetes and Exhibits Altered Diffusion at Disease Onset. *Diabetes Care* **42**, 248-257 (2019).

31. A. Shalev *et al.*, Oligonucleotide microarray analysis of intact human pancreatic islets: identification of glucose-responsive genes and a highly regulated TGFbeta signaling pathway. *Endocrinology* **143**, 3695-3698 (2002).

32. J. Chen *et al.*, Thioredoxin-interacting protein deficiency induces Akt/Bcl-xL signaling and pancreatic beta-cell mass and protects against diabetes. *FASEB J* **22**, 3581-3594 (2008).

33. J. Chen, G. Saxena, I. N. Mungrue, A. J. Lusis, A. Shalev, Thioredoxin-interacting protein: a critical link between glucose toxicity and beta-cell apoptosis. *Diabetes* **57**, 938-944 (2008).

34. A. H. Minn, C. Hafele, A. Shalev, Thioredoxin-interacting protein is stimulated by glucose through a carbohydrate response element and induces beta-cell apoptosis. *Endocrinology* **146**, 2397-2405 (2005).

35. G. Xu, J. Chen, G. Jing, A. Shalev, Thioredoxin-interacting protein regulates insulin transcription through microRNA-204. *Nat Med* **19**, 1141-1146 (2013).

36. S. Jo *et al.*, miR-204 Controls Glucagon-Like Peptide 1 Receptor Expression and Agonist Function. *Diabetes* **67**, 256-264 (2018).

37. G. Xu, J. Chen, G. Jing, A. Shalev, Preventing beta-cell loss and diabetes with calcium channel blockers. *Diabetes* **61**, 848-856 (2012).

38. F. Ovalle *et al.*, Verapamil and beta cell function in adults with recent-onset type 1 diabetes. *Nat Med* **24**, 1108-1112 (2018).

# Attachments

## Attachment 1. Common Terminology Criteria for Adverse Events (CTCAE) Version 5

## Attachment 2. Verapamil SR Prescribing Information
